# Pd Supported on CeO_2_ Nanostructures Prepared by Planetary Ball Milling under a Modified Atmosphere for Catalytic Oxidation of CO

**DOI:** 10.1021/acsanm.5c01769

**Published:** 2025-06-04

**Authors:** Enrique Marín, Xavier Vendrell, Jordi Llorca

**Affiliations:** † Institute of Energy Technologies, Department of Chemical Engineering and Center for Research in Multiscale Science and Engineering, 16767 Universitat Politècnica de Catalunya, EEBE, Eduard Maristany 10-14, 08019 Barcelona, Spain; ‡ Department of Inorganic & Organic Chemistry and Institute of Nanoscience and Nanotechnology (IN2UB), Universitat de Barcelona, 08028 Barcelona, Spain

**Keywords:** ceria, palladium, ball milling, carbon monoxide, mechanochemistry, modified atmosphere

## Abstract

Pd catalysts supported on CeO_2_ (1 wt % Pd) were synthesized using dry mechanochemical and incipient wetness impregnation methods. The mechanochemical synthesis was conducted in a planetary ball mill for 10 min with YSZ milling jar and balls at velocities ranging from 100 to 1000 rpm under various atmospheres (air, N_2_, O_2_, and 5% H_2_/N_2_). Catalysts were evaluated for their performance in the CO oxidation reaction and characterized at the nanoscale using Raman spectroscopy, XPS, XRD, H_2_-TPR, XRF, nitrogen adsorption/desorption, and HAADF-STEM-EDX. Increased catalytic activity was observed in samples milled at lower velocities (100–250 rpm) in air, outperforming that of the conventional sample prepared by impregnation. This improvement appears to be attributed to changes in the surface nanostructure related to the metal–support interaction rather than the presence of oxygen vacancies. XPS revealed a higher concentration of PdO_*x*_-Ce species in the most active milled sample, suggesting this as a reason for its superior catalytic performance. On the other hand, HAADF-STEM-EDX showed the presence of 2–3 nm Pd nanoparticles as well as Pd atoms/clusters anchored on crystalline ceria in the most active milled sample. Samples milled at high velocity (850–1000 rpm) showed a high degree of structural defects in the ceria crystallites and increased Ce^3+^ species, but their catalytic performance was lower than the samples milled at low velocity. This study highlights the impact of milling conditions on the nanostructure and catalytic properties of Pd/CeO_2_ catalysts, with low-velocity milling emerging as a promising approach for enhancing catalytic activity.

## Graphical abstract

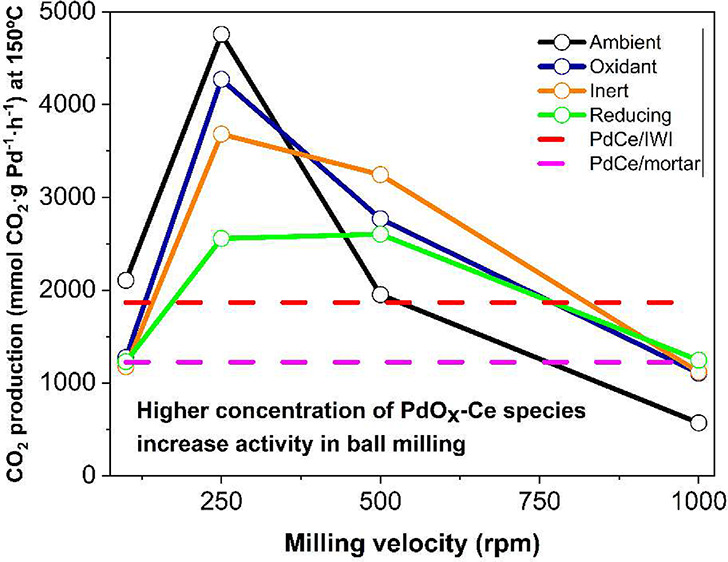

## Introduction

The development of efficient catalysts for carbon monoxide (CO) oxidation is a critical research area with significant environmental and industrial implications. CO is a toxic pollutant primarily produced from incomplete combustion processes in engines, industrial activities, and residential heating systems.^1^ CO inhalation, even in relatively small concentrations, can lead to serious injury, neurological damage and even death.^2^ Eventually, CO takes part in the formation of tropospheric ozone which is one of the major photochemical pollutants of the contemporary world.^3^ Effective oxidation of CO to CO_2_ is essential for reducing air pollution and mitigating health risks associated with CO exposure. Additionally, CO oxidation serves as a model reaction for studying catalytic processes, providing fundamental insights into catalyst behavior, active site characteristics, and reaction mechanisms.^4–6^ Consequently, the pursuit of new and better materials^7^ and novel synthesis methods^8,9^ remains a priority in the field of catalysis.

Recent advances in catalyst development have explored various metals and supports to enhance the performance of CO oxidation catalysts. Transition metals such as platinum, palladium, and rhodium have been extensively studied due to their high catalytic activity.^10–12^ Among these, palladium (Pd) has emerged as a particularly highly effective promoter of active sites on various supports, including ceria, due to its excellent catalytic properties. Pd/CeO_2_ is an active component in emission control catalysts for CO oxidation since Pd enhances the reducibility of ceria, creating highly nanostructured active sites that significantly lower the activation energy for CO oxidation.^13,14^ This synergy between Pd and ceria leads to improved catalytic performance and durability. Additionally, Pd’s resistance to poisoning by contaminants such as sulfur makes it a robust choice for real-world applications.^15^ Different support materials, such as alumina, silica, and titania have also been investigated;^11,16,17^ however, cerium oxide stands out due to its unique redox properties and excellent oxygen storage capacity (OSC).^18,19^ It is renowned for its ability to undergo reversible redox cycles between Ce^3+^ and Ce^4+^ states, which is crucial for maintaining high catalytic activity during CO oxidation. This property allows cerium oxide to release and store oxygen easily, thereby facilitating the dispersion and stabilization of metal nanoparticles and enhance catalytic performance of CO oxidation.^20^ Moreover, cerium oxide’s high thermal stability and resistance to sintering make it an ideal support material for high-temperature catalytic applications^21^ as well as for environmental catalysis.^22^

Mechanochemical synthesis has gained attention as a promising method for catalyst preparation.^23–25^ This technique involves using mechanical force to induce chemical reactions and nanostructural changes in the materials, leading to the formation of highly dispersed and uniformly distributed active phases. Planetary ball mills are specialized grinding machines that consist of one or more grinding jars, which rotate around a central axis while simultaneously rotating on their own axes.^26^ This dual motion creates intense mechanical forces that facilitate the formation of nanoscale structures and enhance the interaction between the active metal and support.^27^ The incipient wetness impregnation (IWI) method, a traditional approach for catalyst preparation, involves impregnating a support material with a solution containing the desired metal precursor, followed by drying and calcination. While widely used, IWI has several disadvantages such as a poor metal dispersion^28^ and environmental impact due to the involvement of solvents.^29^ Given the limitations of IWI, there are high expectations for the application of planetary ball milling in catalyst preparation. This technique is anticipated to produce catalysts with enhanced activity and stability due to better metal–support interaction and improved dispersion of active phases.^30–32^ Atmosphere modification has been previously employed in various material processing methods, illustrating distinct effects depending on the material and environment. For instance, the active role of oxygen has been demonstrated in the mechanical deformation of natural graphite, where oxide formation at active centers generated during the milling process plays a significant role.^33^ In the case of copper nanopowders processed via milling techniques, an inert atmosphere is preferred over air due to its beneficial effects on material properties.^34^ Additionally, the milling atmosphere has been shown to influence both the surface morphology and the functional groups of ball-milled biochar used in the sorption of reactive dyes.^35^

In this work, we compare the performance of Pd/CeO_2_ catalysts prepared by planetary ball milling under different milling atmospheres with that of catalysts obtained with the IWI method, focusing on their nanostructural characteristics, catalytic activity, and stability. By exploring the benefits of mechanochemical synthesis, this research seeks to advance the development of highly efficient catalysts for CO oxidation, contributing to cleaner air, more sustainable industrial processes, and offering insights into the advantages and limitations of this synthesis technique.

## Experimental Section

### Preparation of CeO_2_

Polycrystalline cerium dioxide (CeO_2_) was synthesized through a precipitation method followed by calcination. Cerium­(III) chloride heptahydrate (CeCl_3_·7H_2_O, 99 wt %) was provided by Alfa Aesar and ammonia solution (NH_3_, 28 wt %) from Scharlab. All reagents were used without further purification. To produce the CeO_2_, 180 mL of ammonia solution was gradually added to 1200 mL of 0.15 M cerium­(III) chloride heptahydrate solution while stirring continuously overnight. The precipitate was filtered and extensively washed with deionized water until pH 7 ensuring the removal of any impurities such as NH_4_^+^ and Cl^–^. Subsequently, the precipitate was dried at 110 °C for 12 h and calcined at 500 °C for 4 h with a heating rate of 5 °C/min.

### Preparation of Pd/CeO_2_

The palladium metal loading was fixed to 1 wt % and the Pd precursor used was palladium­(II) nitrate (Pd­(NO_3_)_2_·*x*H_2_O) acquired from Acros Organics and utilized with no further purification. Two preparation methods were used for the preparation of Pd/CeO_2_, namely mechanochemistry (ball milling) and incipient wetness impregnation (IWI), as illustrated in Scheme 1. For the ball milling method, the catalysts were prepared by directly dry milling the metal precursor with CeO_2_ using a planetary ball milling apparatus (Fritsch Pulverisette 7 premium line) at various milling speeds for 10 min (or as specified for 5 and 30 min). The milling process was conducted in an yttrium-stabilized zirconium oxide vessel equipped with gas valves. The ball-to-powder ratio (BPR) was set at 12:1 in all cases, using 10 mm diameter yttrium-stabilized zirconium oxide balls (9 balls), except for milling at 1000 rpm where 5 mm diameter ball were employed (72 balls) following the operational recommendations from the manufacturer. The specific impact energy is proportional to the ball mass and the collision frequency, which depends on the ball size and number of balls used. These two factors compensate each other and reflect a trade-off between impact energy and impact frequency. Ball milled samples are designated as PdCe/BM*x–y*, where *x* denotes the milling speed (100, 250, 500, 850, and 1000 rpm) and *y* represents the atmosphere (A for ambient, R for reducing, O for oxidizing and I for inert). For experiments under modified atmospheres, the respective gases were introduced through the gas valves into the milling jar for 1 h: 5% H_2_/N_2_ for reducing conditions, 100% O_2_ for oxidizing conditions, and 100% N_2_ for inert conditions. Continuous purges were performed during the first minute of gas injection. Samples were tested in the CO oxidation reaction without further treatments. As a control, Pd/CeO_2_ samples prepared at 250 and 500 rpm were calcined at 450 °C for 4 h (5 °C/min), resulting in a significant decrease in catalytic performance in CO oxidation tests (Figure S1). For comparison, Pd/CeO_2_ catalysts were also prepared using the conventional incipient wetness impregnation method, labeled as PdCe/IWI. A specified amount of Pd­(NO_3_)_2_·*x*H_2_O was dissolved in approximately 5 mL of deionized water. This solution was added dropwise to the ceria support with intermittent drying steps at 110 °C for 10–15 min. The sample was then dried at 110 °C for 12 h and calcined at 450 °C for 4 h (5 °C/min). Additionally, a Pd/CeO_2_ catalyst was prepared manually using a pestle and mortar for 10 min at a velocity of about 170–200 rpm, labeled as PdCe/mortar. Furthermore, a sample of CeO_2_ was ball milled at 250 rpm to investigate the impact of milling alone on the support, labeled Ce/BM250-A. The Pd content was measured in all cases via X-ray fluorescence (XRF) with measured values within ± 3% of the nominal composition.

**Scheme 1:**
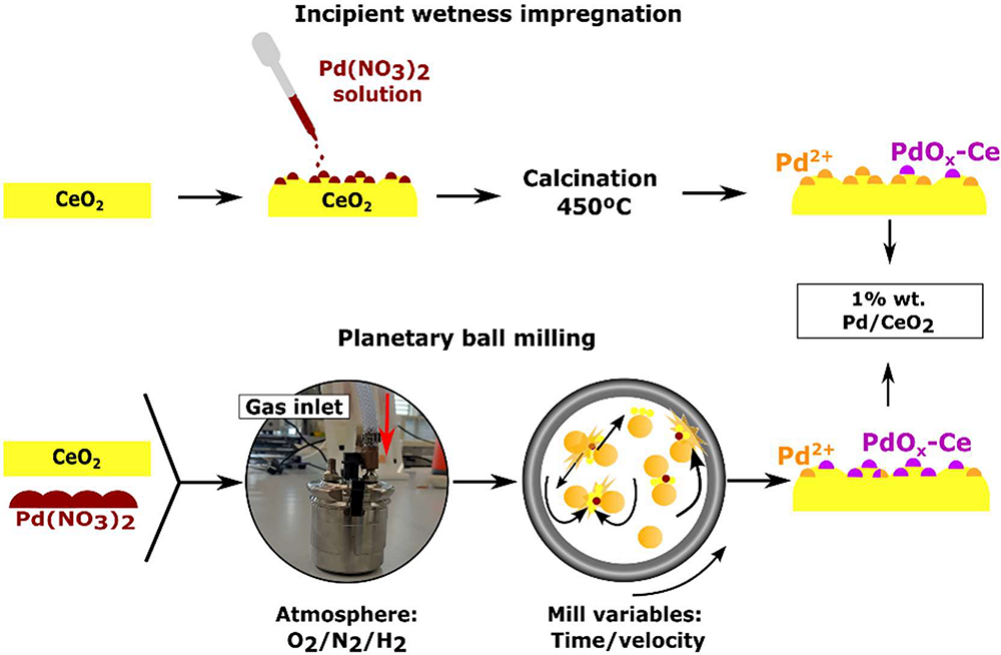
Illustration of the Two Methods Used to Prepare the Pd/CeO_2_ Samples: Incipient Wetness Impregnation and Planetary Ball Milling with Atmosphere Control

### Characterization of Catalysts

Raman spectra were obtained using a confocal Raman spectrometer (Renishaw in Via Qontor) employing a Leica DM2700 M microscope (magnification 50x) with a nominal 100 mW output power. Spectra were typically collected with a 532 nm laser excitation and a 2400 lines mm^–1^ grating over the range of 15–1280 cm^–1^ under ambient conditions. The laser power was adjusted to 1% of its nominal output power, the exposure time was 5 s, and spectra were collected with 5 accumulations. The intensity of all spectra was normalized to the dominant peak corresponding to the symmetrical stretching mode of the ceria fluorite phase (F_2g_ mode) located at 462 cm^–1^. Background subtraction was applied for calculating the *I*_D_/*I*_F2g_ ratio only. X-ray powder diffraction (XRD) of the samples was carried out in a PANalytical X’Pert PRO MPD α1 powder diffractometer configured in Bragg–Brentano geometry. The instrument utilized a Ni-filtered Cu Kα radiation source (λ = 1.5418 Å, 40 mA, 45 kV) with 0.04 radians. Diffraction patterns were recorded over a 2θ ranging of 4.5–100° with a step size of 0.026° and a dwell time of 10 s per step. Crystallite sizes (τ) were determined using the Scherrer equation 1).

$$\tau =\frac{k\lambda }{\beta \cos\theta }$$ 1

where, *k* = 0.9 (assuming the grain shape is spherical), λ is the wavelength (Å), β is the full width at half-maximum (rad) after subtraction of instrumental effects, and θ is the Bragg angle (rad). The lattice parameter of CeO_2_ was calculated using the formula derived from Bragg’s Law and the geometry of a cubic crystal system. The lattice parameter and crystallite size were calculated for the (111), (200), (220), and (311) reflections fitted using a nonlinear Voigt profile function and the results were averaged. Elemental analysis of the catalysts was performed with an PANalytical B Epsilon 3 X-ray fluorescence energy dispersion spectrophotometer (EDXRF). X-ray photoelectron spectroscopy (XPS) was conducted on a SPECS system equipped with a PHOIBOS 150 EP hemispherical energy analyzer and a MCD-9 detector, utilizing an Al Kα X-ray source (1486.6 eV) at 150 W power. A flood gun operating at 10 μA and 1.5 eV was used for charge compensation. Binding energy values were calibrated using the C 1s peak at 284.8 eV for all elements, except for cerium, which was calibrated using the u_3_ Ce 3d peak at 916.9 eV.^7,36^ Prior to quantitative analysis, the Ce 3d, Pd 3d and O 1s core-level spectra were deconvoluted based on established binding energies and fitting parameters. A Shirley-type background was subtracted from each spectrum, and all spectra were fitted using a product of Lorentzian and Gaussian functions. Atomic fractions were calculated using relative sensitivity factors (RSFs) from Casa XPS database and peak areas normalized on the basis of acquisition parameters after background subtraction. The Ce 3d spectra are deconvoluted into ten peaks corresponding to five pairs of spin–orbit doublets, where u and v refer to the 3d_3/2_ and 3d_5/2_ spin–orbit components, respectively.^37^ The peaks assigned to Ce­(IV) are labeled as *u*_3_ (916.9 eV), *v*_3_ (898.4 eV), *u* (901.0 eV), *v* (882.5 eV), *u*_2_ (907.3 eV), and *v*_2_ (889.0 eV), while the peaks *u*_1_ (902.7 eV), *u*_0_ (899.0 eV), *v*_1_ (884.2 eV), and *v*_0_ (880.6 eV) are assigned to Ce­(III). Generally, the concentration of Ce^3+^ = Ce^3+^/(Ce^3+^+Ce^4+^) ratio is indicative of the concentration of surface oxygen vacancies.^38^ Hydrogen temperature-programmed reduction (H_2_-TPR) experiments were performed using a Micromeritics AutoChem II 2920 chemisorption apparatus. Approximately 50 mg of catalyst was pretreated at 150 °C (10 °C/min) under an Ar flow (50 mL/min) for 1 h, then cooled to 25 °C. The temperature was then ramped from 25 to 825 °C (10 °C/min) under a 10% H_2_ in Ar flow (50 mL/min) with a hold time of 30 min at 825 °C. The TCD signal was calibrated using copper oxide (CuO, Panreac, 97%). Textural properties were investigated through nitrogen adsorption/desorption experiments at 77 K using a QUADRASORB evo instrument. The specific surface area was determined using the Brunauer–Emmett–Teller (BET) equation, based on adsorption data within a relative pressure (*P*/*P*_0_) ranging of 0.05–0.25. Total pore volume (*V*_tot_) was calculated at the highest relative pressure (*P*/*P*_0_ = 0.99). Pore size and distribution were assessed utilizing a nonlocal density functional theory (NLDFT) model from the nitrogen adsorption isotherms collected at 77 K. Surface morphology was characterized by scanning electron microscopy (SEM). Micrographs were obtained using a Zeiss Neon40 Crossbeam Station instrument operating at an accelerating voltage of 5 kV and equipped with a field emission electron source. Sample preparation involved depositing a drop of a catalyst suspension in ethanol onto a silicon wafer, followed by air drying. High-angle annular dark-field scanning transmission electron microscopy (HAADF-STEM), high-resolution transmission electron microscopy (HRTEM), and energy dispersive X-ray analysis (EDX) were performed on a JEOL JEM ARM200CF aberration-corrected electron microscope at 200 kV to examine the microstructure of the samples. The average particle diameter was determined from the mean diameter frequency distribution using eq 2), where *n_i_* represents the number of particles with diameter *d_i_* within a specified size range.

$$d(\mathrm{nm})=\frac{\sum n_{i}d_{i}}{\sum n_{i}}$$ 2

### Catalytic Measurements

Carbon monoxide oxidation tests were performed in a fixed-bed continuous-flow reactor made of 316 grade stainless steel (outer diameter 3/8″ and thickness 0.065″), operating at atmospheric pressure. 0.1 g of catalyst was mixed with SiC to form a fixed bed volume of 0.5 cm^3^, centrally placed in the reactor and fixed with inert quartz wool. The feed gas composition was CO:O_2_:N_2_ = 1:1:23, with a flow-to-weight ratio (F/W) of 30 L h^–1^ g^–1^ and a gas hourly space velocity (GHSV) of 6 × 10^3^ h^–1^. The reactor’s temperature was increased from room temperature (RT) to 300 °C in steps of 15 or 20 °C using an electric furnace regulated by a proportional-integral-derivative (PID) temperature controller. Each temperature step involved a dwell time of 90 min to ensure steady-state conditions. No preactivation of the catalyst was done prior to the reaction. The composition of reactants and products was measured online with a gas chromatograph (Agilent Technologies 3000A) equipped with a 5 Å molecular sieve and a PoraPlotU column. CO conversion was calculated using eq 3):

$$\mathrm{CO}\;\mathrm{conversion}\;(\%)=\left(\frac{[\mathrm{CO}{]}_{\mathrm{in},\mathrm{vol}\%}-[\mathrm{CO}{]}_{\mathrm{out},\mathrm{vol}\%}}{[\mathrm{CO}{]}_{\mathrm{in},\mathrm{vol}\%}}\right)\times 100=(\frac{[{\mathrm{CO}}_{2}{]}_{\mathrm{out},\mathrm{vol}\%}}{[{\mathrm{CO}}_{2}{]}_{\mathrm{end},\mathrm{vol}\%}})\times 100$$ 3

where [CO]_in,vol%_, [CO]_out,vol%_, are the inlet and outlet volume concentrations of CO, [CO_2_]_out,vol%_ is the outlet volume concentration of CO_2_, and [CO_2_]_end,vol%_ is the outlet volume concentration of CO_2_ at the end of the reaction. The temperature corresponding to 20, 50 and 90% CO conversion (*T*_20_, *T*_50_ and *T*_90_) were determined assuming linearity with eq 4):

$$T_{c}=\frac{\left(x_{c}-y_{1}\right)\left(x_{2}-x_{1}\right)}{y_{2}-y_{1}}+x_{1}$$ 4

where *T_c_* is the temperature at c conversion (°C), *x_c_* is the desired conversion (%), *x*_1_ and *x*_2_ are the conversions just before and after *x_c_* (%), and *y*_1_ and *y*_2_ are the corresponding temperatures (°C). The absolute error (*E*_abs_) in these temperature measurements was calculated using eq 5):

$$E_{\mathrm{abs}}=\frac{\mathrm{STD}}{\sqrt{n}}$$ 5

where STD is the standard deviation and n is the number of repetitions (in this study, *n* = 3). Long-term stability tests were performed by maintaining the sample at 150 °C for 70 h under the reaction atmosphere. Additionally, thermal cycling tests were performed by subjecting the sample to three cycles of heating and cooling between room temperature (RT) and 200 °C at a rate of 2 °C min^–1^ under the reaction atmosphere.

### Computational Design of Experiments (DOE)

The Taguchi method is a statistical approach used to optimize process parameters and improve quality through robust design. It employs orthogonal arrays to systematically investigate multiple factors using a reduced number of experiments. The method uses the signal-to-noise (S/N) ratio to evaluate performance characteristics and identify optimal conditions by minimizing variability and maximizing the desired response. In this study, a L16 (4^2^) orthogonal array was used within the framework of the Taguchi method to systematically investigate the effects of milling atmosphere and milling velocity on the catalytic performance of Pd/CeO_2_ catalysts in CO oxidation. The design included two factors, each at four levels: milling atmosphere (ambient, reducing, oxidizing, and inert) and milling velocity (100, 250, 500, and 1000 rpm), as detailed in Table S1. This resulted in 16 experimental runs, covering all possible combinations of the selected factor levels (Table S2). By exploring the entire parameter space, the full factorial design provided a comprehensive data set that captured both the main effects of the factors and their interactions. This approach was chosen to ensure a comprehensive understanding of how milling conditions influence catalytic activity, providing robust insights into the optimal preparation conditions for Pd/CeO_2_ catalysts. To identify the optimal conditions that are robust to variations, signal-to-noise (S/N) ratios were selected as the optimization criterion. The S/N ratios for “the larger the better” situations were calculated for CO_2_ production by the following eq 6):

$$S/N\;\mathrm{ratio}(\eta )=-10\times \log\left(\frac{1}{n}\sum_{i=1}^{n}\frac{1}{y_{i}^{2}}\right)$$ 6

where *n* represents the number of experimental iterations, while *y_i_* corresponds to the observed output, specifically the average of CO_2_ production (mmol CO_2_·g Pd^–^^1^·h^–1^) at three temperatures: 115, 150, and 180 °C. The CO_2_ production was calculated using the following equation 7):

$${\mathrm{CO}}_{2}\;\mathrm{production}\left(\frac{\mathrm{mmol}{\mathrm{CO}}_{2}}{\mathrm{gPd}\cdot h}\right)=\frac{xQ_{\mathrm{CO}}}{w_{\mathrm{Pd}}}$$ 7

where *x* represents the CO conversion, *Q*_CO_ is the incoming CO molar flow rate in mmol·h^–1^ and *w*_Pd_ is the weight of the metal in the catalyst in grams.

## Results and Discussion

### Influence of Milling Velocity on CO Oxidation Performance

Figure 1 summarizes the *T*_20_, *T*_50_ and *T*_90_ values obtained in the oxidation of CO of Pd/CeO_2_ samples prepared by ball milling at different velocities (between 100 and 1000 rpm) in air as well as the values recorded over the Pd/CeO_2_ samples prepared by incipient wetness impregnation (IWI) and mortar and pestle. The dispersion of three independent experiments is represented by the error bars in the figure.

**Figure 1:**
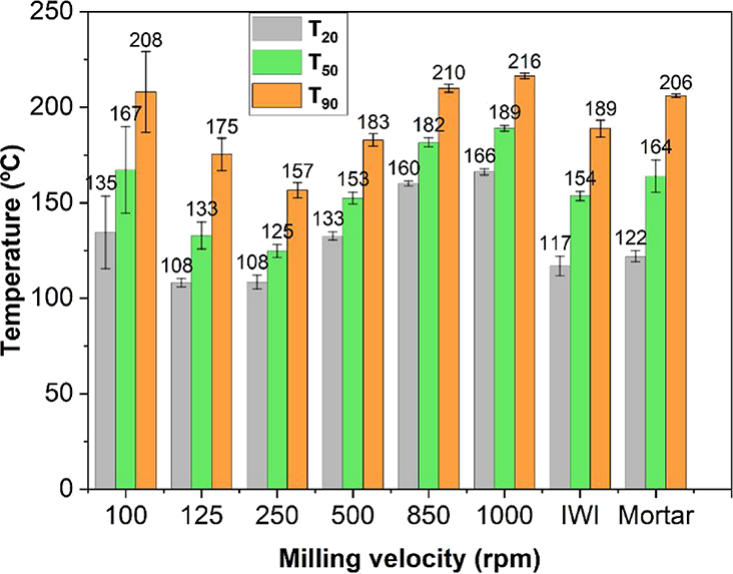
Temperatures for 20, 50 and 90% CO conversion for Pd/CeO_2_ catalysts prepared by incipient wetness impregnation, mortar and pestle, and ball milling at velocities ranging from 100 to 1000 rpm in air.

Notably, the activity of the ball-milled samples is highly dependent on the milling velocity. The best results in terms of catalytic activity are obtained for the sample milled at 250 rpm, with a *T*_50_ value of 125 °C. At milling velocities of 500, 850, and 1000 rpm, the samples show a progressive poorer catalytic activity. At lower milling velocities, 125 and 100 rpm, the samples not only show a poor catalytic performance, but they became less reproducible, as evidenced by a progressive increase of data dispersion corresponding to independent experiments. The absolute errors in *T*_20_, *T*_50_, and *T*_90_ for each sample are compiled in Table S3. This variability in catalytic performance is likely due to a loss of homogeneity in the samples, which may be directly related to the energy imparted to the powders during the milling process. At lower milling velocities, the reduced mechanical energy might result in less uniform particle size distribution and dispersion of active sites, affecting the consistency of the catalytic performance. Thus, there is a delicate balance in optimizing the milling energy to achieve a uniform and highly active catalyst without compromising the homogeneity of the sample. On the other hand, the catalytic performance of the sample milled at 250 rpm outperforms those of the conventional sample prepared by incipient wetness impregnation (*T*_50_ ∼ 159 °C) and the sample prepared manually by grinding with mortar and pestle (*T*_50_ ∼ 164 °C). Overall, these findings highlight the critical role of milling parameters in the mechanochemical synthesis of Pd/CeO_2_ catalysts and their subsequent catalytic performance. By carefully optimizing the milling conditions, it is possible to enhance the catalytic efficiency and reliability of these catalysts for CO oxidation.

### Influence of the Milling Atmosphere on CO Oxidation Performance

To study in detail and systematically the effect of the milling atmosphere and milling energy on the catalytic behavior of the catalysts prepared by ball milling, a set of 16 orthogonal array experiments was designed and executed, with random assignments as detailed in Tables S1 and S2. The experimental results were analyzed using the Taguchi method, employing the signal-to-noise (*S*/*N*) ratio to quantify the influence of each factor. The *S*/*N* ratios were calculated as the average for each level, as presented in Table S4, and the corresponding effects are illustrated in Figure 2A. The effects of these two parameters on the CO oxidation reaction was assessed by quantifying the CO_2_ production at three temperatures: 115, 150, and 180 °C. The results indicate that the milling atmosphere has a measurable impact on CO oxidation activity. Specifically, a reducing atmosphere yielded the lowest activity across the temperature range studied (115–180 °C). In contrast, oxidizing and inert atmospheres exhibited slight enhancements in catalytic performance compared to atmospheric conditions. In terms of milling velocity, a much stronger effect on the catalytic response was observed. The data confirm that a milling velocity of 250 rpm is the optimal condition, resulting in the highest catalytic activity for the CO oxidation reaction. The interaction plot in Figure 2B illustrates the combined effects of milling atmosphere and milling velocity on the catalytic performance. The results reveal that the positive influence of oxidizing and inert atmospheres is more pronounced at higher milling velocities (500 and 1000 rpm), corresponding to high-energy conditions. In contrast, this effect diminishes under low-energy milling conditions. Interestingly, while 250 rpm was identified as the optimal milling velocity for CO oxidation activity, its interaction with oxidizing or inert atmospheres appears to have limited significance compared to atmospheric conditions. This suggests that under low-energy milling, the catalytic activity is predominantly governed by factors other than the atmosphere, highlighting a complex interplay between the two parameters.

**Figure 2:**
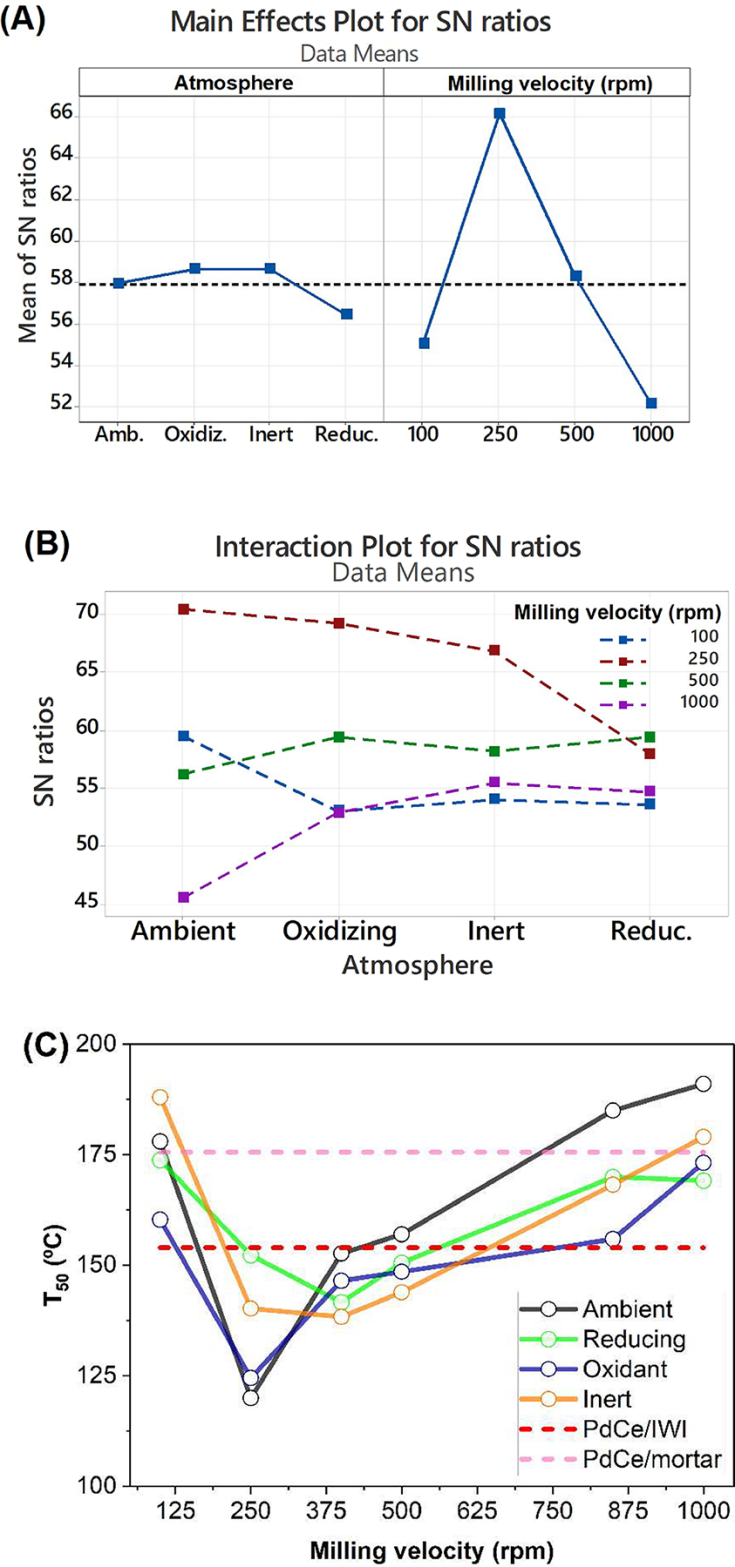
Effect plot (A) and interaction plot (B) of the milling atmosphere and milling velocity on the mean of *S*/*N* ratios. (C) Temperature at 50% CO conversion (*T*_50_) for Pd/CeO_2_ catalysts prepared by ball milling at velocities ranging from 100 to 1000 rpm under different atmospheres. Values for catalysts prepared by IWI and mortar and pestle methods are included as dashed lines.

Figure 2C shows the CO oxidation performance, expressed as the temperature required to achieve 50% CO conversion (*T*_50_). The results indicate that for samples milled at 250 rpm, the highest catalytic activity is observed for those prepared under oxidizing atmospheres (air and pure O_2_). This enhancement is likely attributable to specific metal–support interactions promoted by the oxidative environment during milling. However, when considering other milling velocities, no consistent trends are evident regarding the influence of the milling atmosphere. For comparison, *T*_50_ values of catalysts prepared using traditional methods, including incipient wetness impregnation (IWI) and mortar grinding, are also included. These traditional methods generally show lower performance compared to the ball-milled samples, underscoring the advantages of mechanochemical synthesis. Therefore, among the various conditions tested, the sample milled at 250 rpm in air (PdCe/BM250-A) emerges as the optimal one, offering the best balance between catalytic activity, reproducibility, and simplicity. This finding highlights that moderate milling velocities in an unmodified atmosphere can produce highly active and reliable Pd/CeO_2_ catalysts, leveraging the intrinsic benefits of the mechanochemical method without the need for atmospheric alteration. Thus, it is clear that the use of mechanochemistry for the preparation of Pd/CeO_2_ catalysts for CO oxidation offers numerous advantages, such as higher catalytic activity, energy and time savings compared to more conventional catalyst preparation methods, simplicity, easy control of the atmosphere during synthesis, and the benefit of avoiding the use of environmentally harmful solvents.

### Influence of the Milling Time on CO Oxidation Performance

Figure 3A shows the CO conversion curves of Pd/CeO_2_ catalysts milled at 250 rpm in air for different times (5, 10, and 30 min), as well as bare ceria and a ceria sample milled at 250 rpm in air for 10 min. These samples were compared to those prepared by incipient wetness impregnation (IWI) and mortar and pestle grinding. The catalytic activity of pure ceria and pure ceria milled at 250 rpm is virtually identical, indicating that the low-energy milling process does not introduce defects into the ceria structure that would enhance its catalytic activity. Regarding the milling time for Pd/CeO_2_ milled at 250 rpm, there is a clear dependence with catalytic activity. The best catalytic performance is obtained by milling for 10 min, followed by milling for 30 and 5 min. Again, there is a delicate compromise that highlights the strong influence of milling parameters on the creation of catalytically active sites. At short milling times, the conditions do not allow for an extensive formation of catalytically active sites, whereas large milling times probably destroy the particular surface architectures responsible for high catalytic activity.

**Figure 3:**
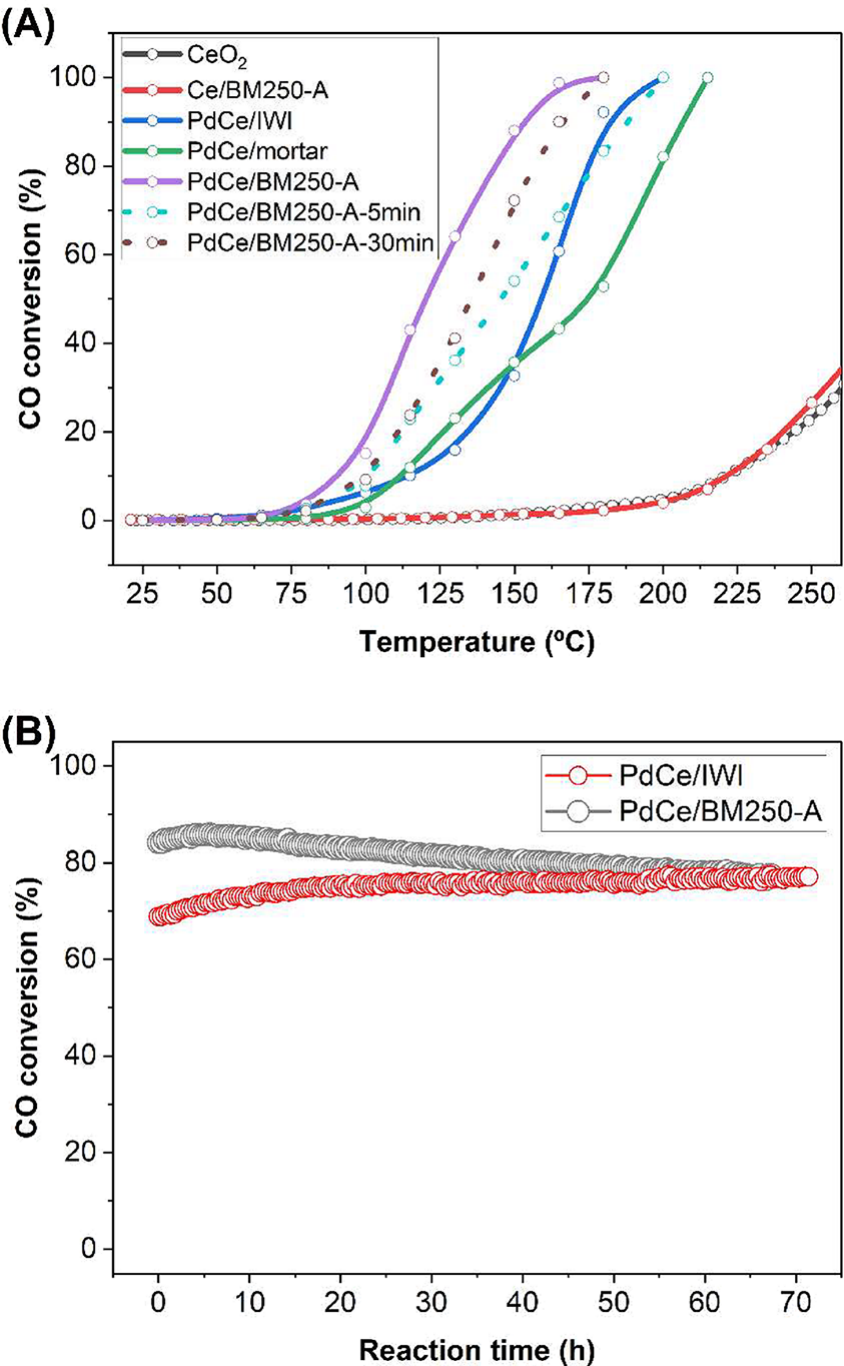
(A) CO conversion curves for bare ceria, ceria milled at 250 rpm in air, and Pd/CeO_2_ catalysts prepared using the mortar and pestle method, incipient wetness impregnation, and ball milling at 250 rpm in air for 5, 10, and 30 min. (B) Long-term stability tests for CO oxidation at 150 °C for Pd/CeO_2_ catalysts prepared by the IWI method and ball milling at 250 rpm for 10 min under air.

### Stability Tests

Long-term stability tests at 150 °C were conducted for 70 h on Pd/CeO_2_ catalysts prepared by incipient wetness impregnation (IWI) and ball milling at 250 rpm in air for 10 min to evaluate their robustness (Figure 3B). The sample PdCe/BM250-A showed an initial catalytic activity significantly higher than that exhibited by the IWI sample; however, it exhibited a gradual decline in activity, ultimately matching the performance level of the PdCe/IWI sample, which showed a slow initial activation until reaching constant stability. To further evaluate the stability of the PdCe/BM250-A and PdCe/IWI catalysts, additional cycling tests were conducted (Figure S2). The consecutive cycles revealed a progressive deactivation of the PdCe/BM250-A sample during the heating ramp, whereas the catalytic activity during the cooling ramp remained constant. Repeated thermal cycling can impact the structural and surface properties of the catalyst, all of which contribute to a decrease in catalytic activity over time. In the case of the IWI sample, the second cycle showed an improved catalytic performance, whereas from the third cycle onward, the activity declined during the cooling ramp.

### Textural Properties

Both bare ceria and Pd-loaded ceria catalysts exhibited type IV adsorption isotherms with characteristic hysteresis loops in the relative pressure range of 0.5–1.0 (Figure S3), which are indicative of mesoporous materials.^39^ The textural characteristics of the catalysts, detailed in Table S5, show that the surface areas of the ball-milled samples are virtually identical to pure ceria at about 50 m^2^ g^–1^ and to the catalyst prepared by impregnation (52 m^2^ g^–1^). The average pore size of the catalysts decreased upon the addition of palladium to ceria. The average pore diameter for bare ceria was 8.9 nm, which decreased to 7.5 nm for all Pd-loaded samples. This phenomenon can be explained by the partial blockage of the pores by Pd clusters, which deposit on the ceria surface and reduce the effective pore diameter.^40^ It is also observed that the different ball milling velocities did not significantly affect the surface area and pore size distribution of the catalysts. However, the total pore volume decreased compared to bare ceria, likely due to the compaction and partial pore collapse induced by the mechanical treatment during ball milling.

### XRD

Figure 4A presents the XRD diffractograms for bare ceria and as-prepared Pd catalysts (IWI, BM250 and BM850). As anticipated, well-defined peaks at 28.5°, 33.0°, 47.4°, 56.3°, 59.1°, 69.4°, 76.6°, 79.0°, 88.3°, and 95.2°, corresponding to the characteristic face-centered cubic (fcc) fluorite-type of ceria, were observed in all samples (JCPDS file card N° 00-34-0394).^41–43^ The intense and narrow diffraction peaks indicate high crystallinity across all samples. Given the low palladium content and its high dispersion on the ceria support, no diffraction lines corresponding to Pd or PdO were detected in the XRD patterns of the Pd-loaded samples. This observation aligns with previous studies where similar Pd loadings did not produce distinguishable Pd-related peaks in XRD analysis due to their nanoscale dispersion.^42,44^ Interestingly, a noticeable peak shift in the CeO_2_ (111) reflection toward higher diffraction angles was observed in the PdCe/BM250-A (Figure 4B), indicative of lattice shrinkage induced by mechanochemical synthesis.^8^ This phenomenon can be attributed to the partial incorporation of Pd species into the ceria lattice, forming a Pd–O–Ce solid solution due to palladium’s smaller ionic radius.^44^ In contrast, the PdCe/BM850-A sample exhibited a peak shift toward lower diffraction angles, which corresponds to an increase in the lattice parameter. This expansion is likely due to the reduction of Ce^4+^ to Ce^3+^, as detected in the Raman spectra (see below), resulting in lattice distortion. The reduction of Ce^4+^ ion leads to an increase in lattice volume due to the larger ionic radius of Ce^3+^ (1.01 Å) compared to Ce^4+^ (0.87 Å).^45,46^ The average crystallite size, calculated using the Scherrer equation, shows a slight decrease in the PdCe/BM850-A sample (Table S6), suggesting that high-energy milling induces a reduction in crystalline domain size. While this observation is consistent with the increased disorder and smaller particle size seen in the HRTEM analysis (see below), we note that TEM particle size is not necessarily equivalent to crystallite size, since particles can be polycrystalline or contain amorphous regions. Therefore, the two measurements are considered complementary indicators of the structural effects of the milling treatment.

**Figure 4:**
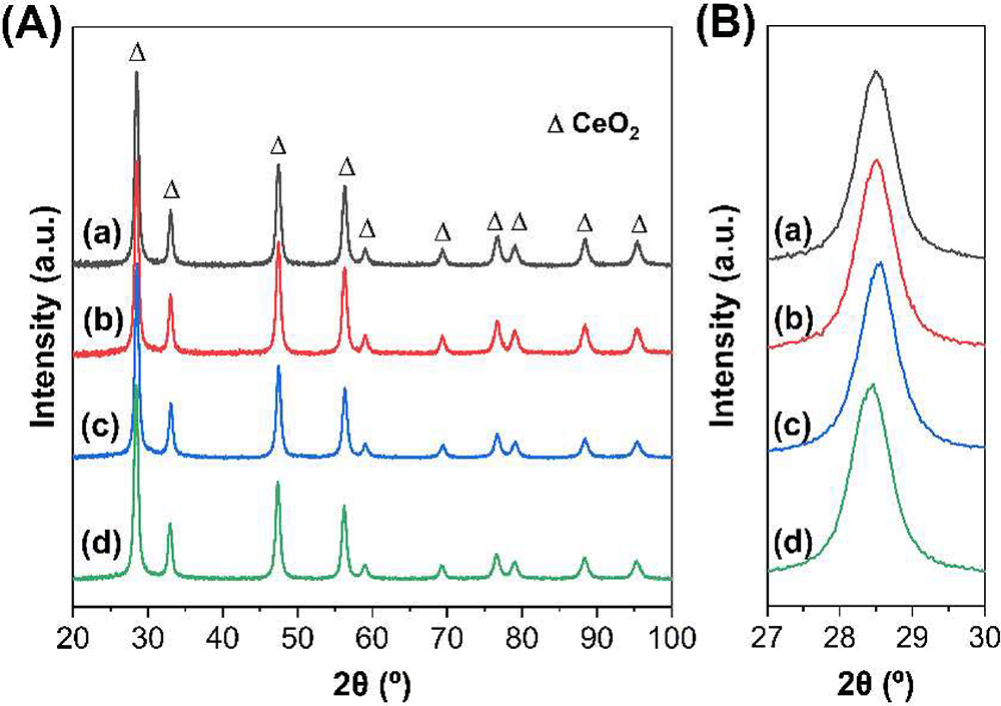
XRD patterns (A) and CeO_2_ (111) close-up (B) of (a) bare ceria, (b) PdCe/IWI, (c) PdCe/BM250-A, and (d) PdCe/BM850-A samples.

### Raman Spectroscopy

The Raman spectra of bare ceria and Pd/CeO_2_ catalysts are depicted in Figure 5. The intensity of all spectra was normalized to the dominant peak corresponding to the symmetrical stretching mode of the ceria fluorite phase (F_2g_ mode) located at 462 cm^–1^.^47^ Three additional weaker peaks were observed at approximately 260, 590–620, and 1170 cm^–1^, which correspond to the second-order transverse acoustic (2TA), the Frenkel defect-induced (D), and the second-order longitudinal optical (2LO) modes of ceria, respectively.^8,48^ The broad features observed in the region around 590–620 cm^–1^ are often referred to collectively as ’D bands’, and are associated with lattice defects such as oxygen vacancies and structural disorder arising from aliovalent substitution (e.g., Ce^3+^, Pd^2+^). The intensity ratio between the defect-related band (*I*_D_) and the F_2g_ mode (*I*_F2g_), presented in Table S6, is used here as a semiquantitative indicator of defect concentration.^8^ However, we emphasize that this ratio must be interpreted cautiously, as the D region may contain contributions from multiple defect types and does not provide a direct quantification of Ce^3+^ content. The data indicate that oxygen vacancies are present in all catalysts, with the PdCe/IWI and PdCe/BM850-A samples showing a significant increase in vacancies compared to bare ceria. Conversely, the PdCe/BM250-A sample, milled at a lower energy velocity (250 rpm), exhibited a reduced Ce^3+^ amount. Additionally, the PdCe/BM850-A sample displayed a blue-shift of the D-band, likely due to the lattice defects resulting from the high energy input during milling. Residual nitrate species were detected in the ball-milled samples, with peaks around 730 and 1045 cm^–1^ corresponding to the υ_4_ and υ_1_ bands of NO_3_^–^ ^49,50^ (Figure S4). This was expected as no calcination was performed after milling. Also, bands between 900 and 1000 cm^–1^ in PdCe/BM850-A sample are attributed to modified nitrate species created by high-energy ball milling, as shown in Figure S5. The nitrate species were completely removed after calcination in the PdCe/IWI sample. Regarding Pd species, a weak peak at 650 cm^–1^ was detected in the IWI sample, which may be assigned to the B_1g_ vibrational mode of PdO.^51^ The F_2g_ band shifted from 462.2 cm^–1^ in bare ceria to 463.3 cm^–1^ in all catalysts, indicating a distortion of the ceria lattice due to the formation of metal–Ce bonds (Figure S6). The ball-milled samples exhibited bands around 305 and 341 cm^–1^, and although no clear assignment could be found, they have been previously reported in the literature for ceria nanoparticles as a strong indicator of the presence of defects,^52^ an intimate interaction between the CeO_2_ support and metals^49^ or distortion in the lattice.^7^ Additionally, a feature at 830 cm^–1^ was detected in high-energy milled samples in modified atmosphere (850 and 1000 rpm, Figure S4B) corresponding to the presence of peroxide species (O–O stretching vibration),^53^ probably associated to lattice defects. No zirconia contamination was detected in the ball-milled samples, as the main peaks at 261 and 642 cm^–1^ (Figure S7) were absent in the Raman spectra. This observation is consistent with the XRF and XPS analyses (Figure S8), which did not report zirconium.

**Figure 5:**
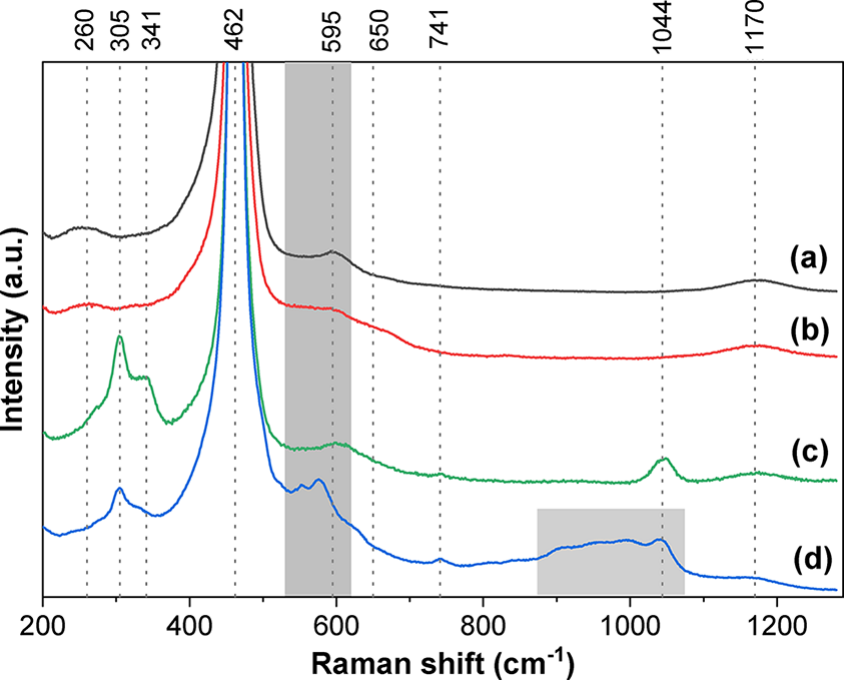
Raman spectra of bare ceria (a) and Pd/CeO_2_ catalysts prepared by the IWI method (b), and ball milling in air at 250 rpm (c), and 850 rpm (d).

To evaluate the formation of carbonaceous deposits on Pd/CeO_2_ catalysts after the reaction, Raman spectroscopy was performed on the PdCe/BM250-A sample following the 70-h stability test and on the PdCe/IWI sample after the thermal cycling test (Figure S9). The results reveal significant differences in the extent of carbon deposition and the nature of surface species between the two preparation methods. For the impregnated catalyst (PdCe/IWI), several highly intense Raman peaks were detected, indicating substantial carbon deposition during the reaction. Specifically, the D band (∼1348 cm^–1^) corresponding to C=C stretching modes in disordered graphitic carbonaceous species, and the G band (∼1640 cm^–1^) related to graphitized carbon.^54,55^ The G band, typically observed around 1580 cm^–1^, may be shifted in this case due to the overlap with a second band, D_2_ (∼1620 cm^–1^).^56^ These carbonaceous deposits likely result from incomplete CO oxidation or the accumulation of carbonaceous intermediates over the course of the reaction. Such deposits are known to adversely affect the long-term catalytic stability of the material by potentially deactivating the catalyst. However, it has been suggested in other studies that the accumulation of carbonaceous species on the ceria surface could indicate an increased presence of Ce^3+^ sites, which might enhance certain catalytic properties.^4^ Additional peaks observed in the Raman spectrum suggest the formation of other surface species at 1476, 1642, and 1790 cm^–1^ potentially associated with surface carbonates, carboxylates, or other reaction intermediates adsorbed on ceria or Pd during reaction. In contrast, the BM250-A catalyst exhibited significantly weaker Raman peaks in the same region, indicating minimal carbon deposition. An inset in the Raman spectra revealed faint peaks at approximately 1180 cm^–1^ which is assigned to the 2LO vibrational mode of ceria, 1340 cm^–1^ and 1580 cm^–1^, corresponding to the D and G bands, respectively, indicating trace amounts of disordered and graphitic carbon. The lower intensity of these peaks for the ball-milled sample underscores its enhanced resistance to carbon deposition, enabling it to sustain catalytic performance over extended operational periods due to its robust structural and chemical stability.

### XPS

The surface composition and chemical states of the catalysts were analyzed using X-ray photoelectron spectroscopy (XPS). Figures 6 and 7A show the Ce 3d, O 1s, and Pd 3d XP spectra for bare ceria, Pd/CeO_2_ prepared by impregnation, and ball-milled samples at 250 and 850 rpm in air. The Ce^3+^/(Ce^3+^ + Ce^4+^) ratio is indicative of the concentration of oxygen vacancies. As shown in Table 1, the ball-milled samples exhibited a decrease in surface Ce^3+^ content compared to bare ceria and PdCe/IWI. The O 1s spectra revealed three distinct oxygen species in all samples. The primary peak around 529.5 eV corresponds to lattice oxygen (O–Ce^4+^), the peak around 531.7 eV is associated with chemisorbed oxygen on Ce^3+^ sites (defects, O–Ce^3+^) or O from PdO_*x*_ species, and the peak around 534.0 eV is assigned to hydroxyl groups.^44,57^ Clearly, the PdCe/BM250-A sample exhibited a significantly lower O–Ce^3+^/total O ratio than the other samples, indicating that the presence of oxygen vacancies and/or Ce^3+^ is not directly correlated with the higher catalytic activity observed.

**Table 1:** Palladium Content Measured by XRF and Surface Elemental Composition Measured by XPS^a^

Sample	Pd (wt %)^a^	Ce^3+^/Ce (%)	O–Ce^3+^/Total O–Ce (%)	Pd^2+^ (%)	PdO_*x*_-Ce (%)	Pd/Ce	T_50_ (°C)
CeO_2_	0.00	33	22	0	0	---	301
PdCe/IWI	0.97	37	19	81	19	0.060	159
PdCe/BM250-A	1.02	26	13	24	76	0.059	120
PdCe/BM850-A	0.95	29	18	55	45	0.065	185

a The T_50_ values in the oxidation of CO are included for comparison.

b Obtained by XRF.

**Figure 6:**
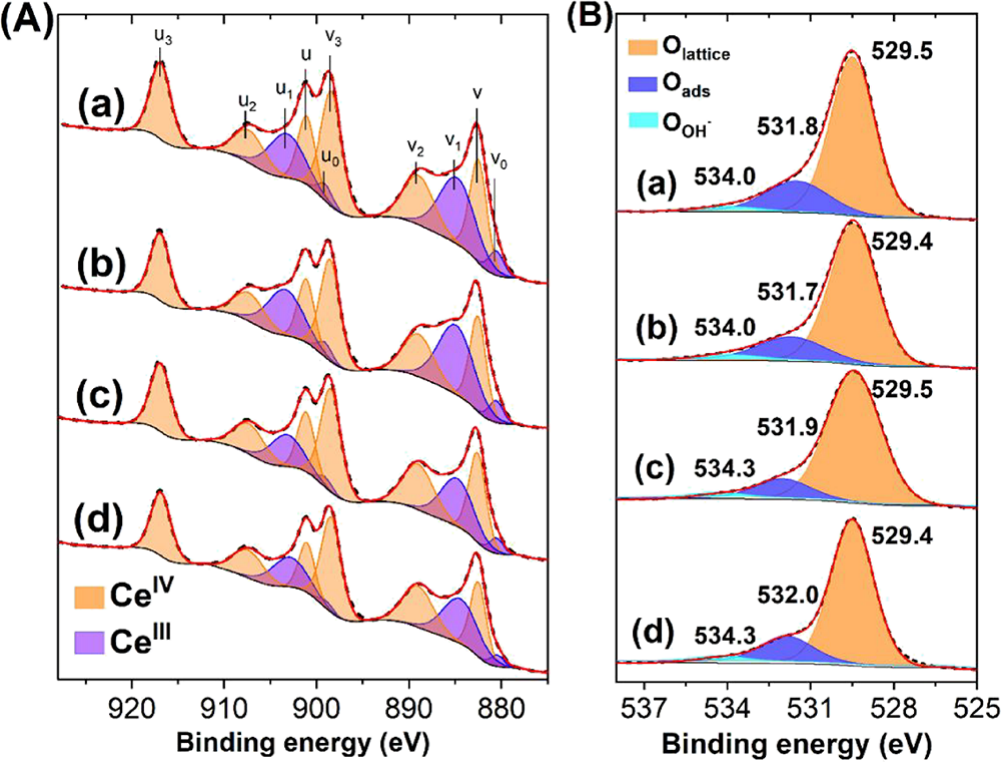
Ce 3d (A) and O 1s (B) X-ray photoelectron spectra of bare ceria (a) and Pd/CeO_2_ catalysts prepared by incipient wetness impregnation (b) and ball milling in air at 250 rpm (c) and 850 rpm (d).

**Figure 7:**
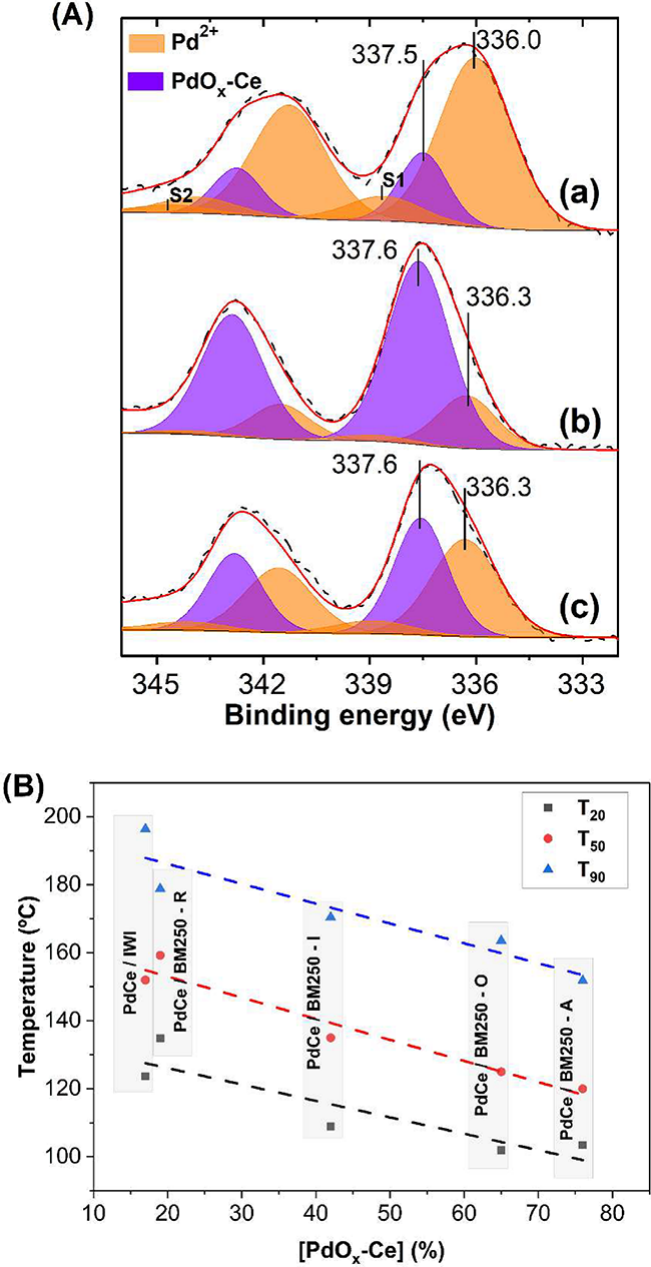
(A) Pd 3d X-ray photoelectron spectra of Pd/CeO_2_ catalysts prepared by incipient wetness impregnation (a) and ball milling in air at 250 rpm (b) and 850 rpm (c). (B) Temperature at 20, 50 and 90% of CO conversion versus the concentration of PdO_*x*_-Ce species for impregnated and milled samples at 250 rpm under different atmospheres.

The surface Pd/Ce atomic ratios calculated from XPS indicated that the distribution of palladium on the ceria surface was similar across all samples (0.059–0.065), which is significantly higher than the nominal bulk atomic ratio of 0.016 for a 1 wt % Pd/CeO_2_ catalyst. The Pd 3d spectra exhibited notable differences between the mechanochemically prepared samples and the conventionally impregnated sample. The Pd 3d_5/2_ spectra displayed two bands at approximately 336.0 and 337.5 eV, ascribed to Pd^2+^ and PdO_*x*_-Ce species, respectively.^58^ In addition, two satellite doublets were included in the fitting (S1 and S2), corresponding to shakeup features characteristic of Pd^2+^ species such as PdO. The S2 satellite is attributed to charge transfer processes involving Pd, while the origin of the S1 satellite remains under discussion.^59^ Their contribution was incorporated into the analysis of the surface composition. The proportion of PdO_*x*_-Ce species increases markedly in the ball-milled samples, from 19% for the PdCe/IWI sample up to 76% in the PdCe/BM250-A catalyst. Therefore, the superior catalytic activity of the catalysts prepared by ball milling is ascribed to the existence of surface, nanostructured PdO_*x*_-Ce species. To support this further, Figure 7B shows the strong relationship existing between the T_20_, T_50_ and T_90_ values recorded over the PdCe/BM250 catalysts prepared under different atmospheres and the amounts of PdO_*x*_-Ce species obtained from their XP spectra. PdO_*x*_-Ce nanostructures have been recognized as highly active sites in other oxidation reactions, such as methane total oxidation.^55^ Clearly, the higher the amount of surface PdO_*x*_-Ce the higher the catalytic activity (see Figure S10 for Pd 3d spectra under different atmospheres and Table S7).

XPS analyses were conducted on the samples after the 70-h stability test for both BM250-A and IWI samples. Figure S11A presents the Ce 3d XPS spectrum, showing no significant change in the Ce^3+^ content. For the IWI sample, the Ce^3+^ proportion remained constant around 45%, same for the BM250-A sample, it remained stable at approximately 37%. Figure S11B illustrates the Pd 3d spectra, revealing a substantial increase in PdO_*x*_-Ce nanostructures for the IWI sample, rising from 19 to 73%. This pronounced increase may explain the high stability and activity observed in its catalytic performance. In contrast, the PdO_*x*_-Ce content in the BM250-A sample remained relatively constant at around 75%. Notably, the Pd^2+^ peak shifted to lower binding energies (from 336.0 to 335.6 eV), which could be associated with enhanced metal–support interactions or a change in the local electronic environment of Pd species. A stronger Pd–Ce interaction can result in partial electron transfer from the ceria support (particularly from Ce^3+^ sites or oxygen vacancies) to the Pd species, as previously reported in the literature.^60,61^ This electron donation changes the electronic structure of Pd, increasing the electron density around Pd^2+^, thus reducing its core-level binding energy. Such interactions often involve the participation of Ce^4+^ sites or lattice oxygen, which can be partially reduced to Ce^3+^, although this is not reflected by an increased Ce^3+^/Ce^4+^ ratio (Figure S11A).^62^ Such a shift might indicate stronger bonding between Pd and Ce in the BM250-A sample, potentially contributing to its maintained activity over extended reaction times. These findings highlight the differing mechanisms of stabilization in the two catalyst types, with the IWI sample benefiting from an increased formation of PdO_*x*_-Ce species and the BM250-A sample relying on its robust initial Pd–Ce interactions. Regarding the slight deactivation of the ball-milled sample observed during the long-term stability test, and considering that no significant carbon accumulation is detected in the Raman spectrum after the reaction, and that XPS analysis reveals no notable changes in the Ce­(III) concentration or PdO_*x*_-Ce content, we hypothesize that the slightly higher deactivation rate of the milled sample may be associated with dynamic structural rearrangements at the Pd–ceria interface occurring during the reaction.

### SEM, HRTEM, and HAADF-STEM-EDX

The morphology of the samples was characterized by scanning electron microscopy (SEM) both prior to and following the incorporation of palladium (Pd). The SEM micrographs indicate that the ceria polycrystals exhibit an irregular rounded morphology, consistent with previous reports,^7^ with particle sizes ranging from approximately 5 to 20 nm, as depicted in Figure S12. The introduction of Pd, as well as the preparation method employed, did not alter the morphology of the ceria polycrystals.

The nanostructure and elemental distribution of the Pd/CeO_2_ catalysts was studied using high-angle annular dark-field scanning transmission electron microscopy (HAADF-STEM) and energy dispersive X-ray analysis (EDX). Representative images are provided in Figure 8. The analysis of the PdCe/IWI catalyst confirms that the ceria crystals exhibit an average size of 14.7 ± 5.1 nm. In comparison, the PdCe/BM250-A and PdCe/BM850-A samples display slightly smaller average sizes of 12.8 ± 3.7 nm and 11.2 ± 3.1 nm, respectively, consistent with the XRD results discussed above. Additionally, for the PdCe/IWI sample, the analysis reveals a relatively good distribution of Pd entities over the ceria support (Figure 8A). The particle size of Pd ranges from ca. 5 to 15 nm. In contrast, the samples prepared by ball milling contain a better distribution of Pd over the ceria support, mainly below 5 nm in size, although a few particles measuring up to 15–20 are also identified in the catalysts milled at high energy (Figure 8B). High-resolution HAADF-STEM images of the PdCe/BM250-A sample are depicted in Figure 9. In Figure 9A, Pd nanoparticles, which are marked inside red circles, measure 2–3 nm in size and are well anchored on the ceria support crystallites. Figure 9B shows the presence of Pd atoms/clusters on a (200) facet of a ceria particle oriented along the [001] crystallographic direction. The presence of such small Pd nanoensembles can be tentatively correlated with the PdO_*x*_-Ce species identified by XPS in the samples prepared by mechanochemistry and explains the high reactivity of this catalyst in the oxidation of CO.

**Figure 8:**
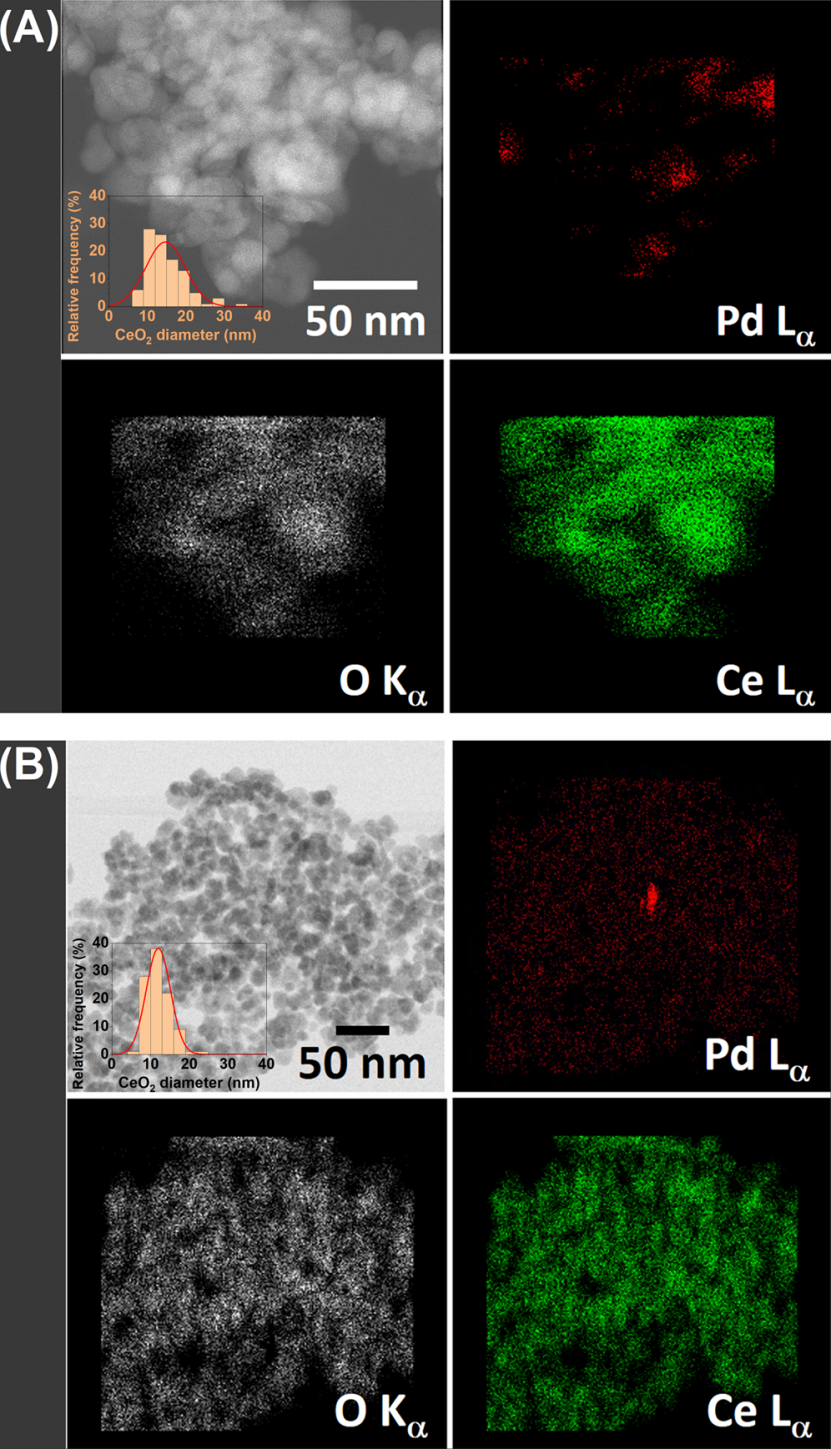
HAADF-STEM-EDX for Pd/CeO_2_ catalysts prepared by (A) incipient wetness impregnation and (B) ball milling at 850 rpm under ambient atmosphere. The insets correspond to particle size histograms of CeO_2_.

**Figure 9:**
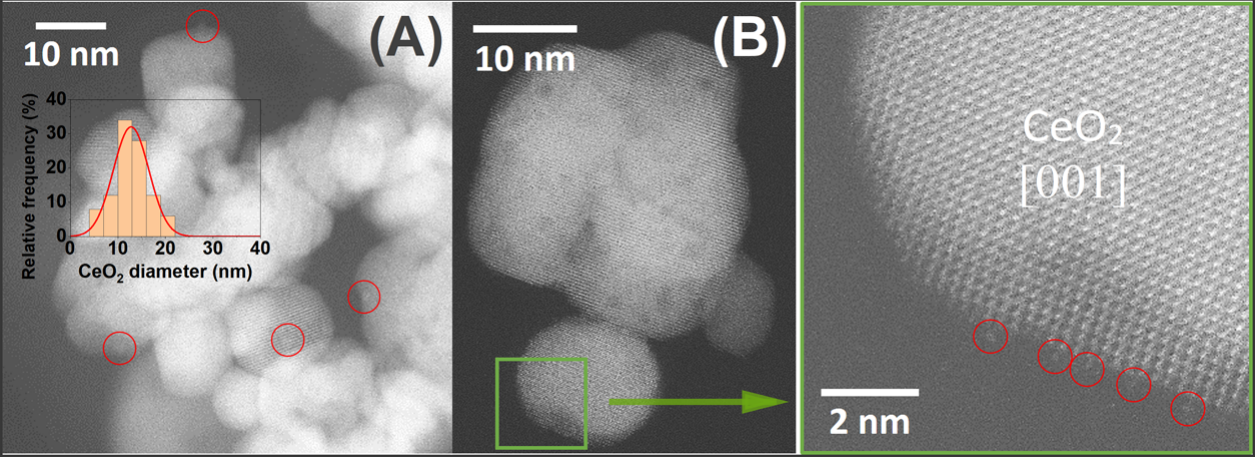
HAADF-STEM images of the Pd/CeO_2_ catalyst synthesized by ball milling at 250 rpm in air. (A) Low-magnification image showing Pd nanoparticles marked inside red circles. The inset corresponds to particle size histogram of CeO_2_. (B) High-magnification image showing Pd atoms/clusters.

Whereas the PdCe/BM250-A catalyst contains well crystallized ceria particles, the HRTEM images recorded over the PdCe/BM850-A sample reveal a pronounced lattice disorder within the CeO_2_ structure, consistent with the high-energy milling conditions employed during the synthesis (Figure S13). This disorder is associated with a substantial presence of defects, as corroborated by Raman spectroscopy results (Figure 5). Notably, this nanostructural disruption does not correlate with the formation of oxygen vacancies or an increase in Ce^3+^ species on the surface, as XPS analysis shows no corresponding rise in these features in the milled samples. Indeed, high-energy ball milling conditions result in the degradation of the active PdO_*x*_-Ce species, as supported by the XPS analysis. This indicates the existence of an optimal range of ball milling conditions necessary for the formation and stabilization of the catalytically active PdO_*x*_-Ce structures. While their generation relies on the mechanochemical process, excessive milling energy can disrupt their specific architecture, ultimately leading to a loss of catalytic activity.

### H_2_-TPR

Figure 10 presents the H_2_-TPR analyses of CeO_2_ and Pd/CeO_2_ catalysts. The reduction profile of bare CeO_2_ typically exhibits a bimodal shape with a first peak at around 475 °C, which corresponds to the reduction of surface Ce^4+^, and a second peak at around 682 °C, which is associated with the reduction of bulk Ce^4+^.^19,63^ In all samples, the bulk CeO_2_ reduction peak remains consistent around 682 °C, indicating that the bulk structure of ceria remains largely unaffected by the presence of Pd, regardless of the preparation method. However, a notable decrease in the surface CeO_2_ reduction temperature is observed across the Pd-containing samples, as well as an increase in the H_2_ consumption values as detailed in Table S8. For pure CeO_2_, this peak appears at 475 °C (230 μmol H_2_/g), whereas it shifts to lower values at approximately 305 °C for PdCe/IWI (1119 μmol H_2_/g), 312 °C for PdCe/BM850-A (1316 μmol H_2_/g), and 335 °C for PdCe/BM250-A (1518 μmol H_2_/g). Palladium incorporation significantly enhances the surface reducibility of ceria through hydrogen spillover from Pd species to the support, as already reported,^44^ leading to a weakening of Ce–O bonds and facilitating oxygen release, which can be then utilized in CO oxidation.^8,64^ The peaks observed between 127 and 187 °C are attributed to the reduction of palladium. Specifically, the PdCe/IWI sample shows a reduction temperature of 127 °C and a relatively low H_2_ uptake (565 μmol H_2_/g), suggesting a weak interaction between Pd and CeO_2_. In contrast, the mechanical energy input in the ball-milled samples results in a stronger Pd-CeO_2_ interaction.^65^ This is especially evident in the PdCe/BM250-A sample, which exhibits the highest reduction temperature (187 °C) and the highest H_2_ uptake (1342 μmol H_2_/g). Similarly, PdCe/BM850-A also shows improved metal–support interaction (153 °C, 1234 μmol H_2_/g). The increased H_2_ uptake in ball-milled samples arises from the strong Pd-CeO_2_ interaction, which facilitates the reduction of neighboring CeO_2_ species at the surface in addition to the Pd.

**Figure 10:**
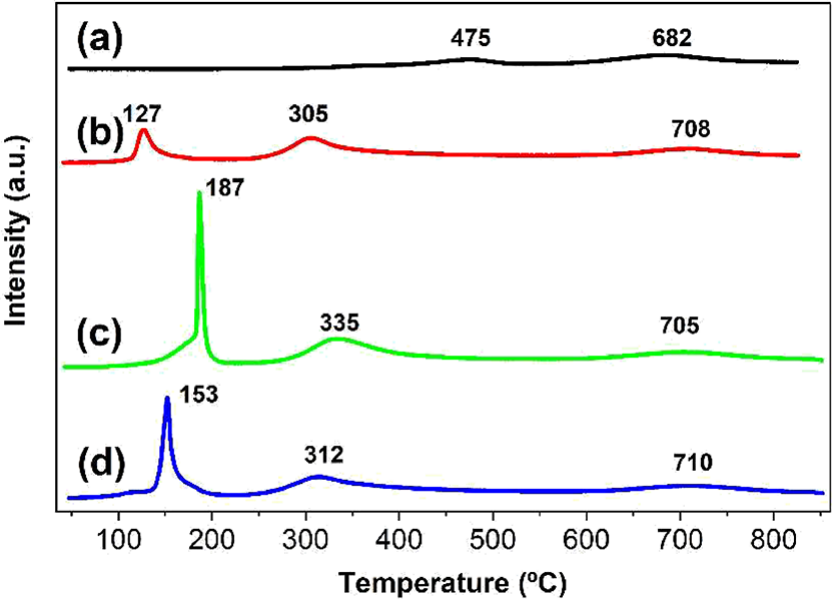
Temperature-programmed reduction (H_2_-TPR) profiles of (a) bare ceria and Pd/CeO_2_ catalysts prepared by (b) incipient wetness impregnation and ball milling at (c) 250 rpm and (d) 850 rpm in air.

## Conclusions

In summary, we have demonstrated that palladium catalysts supported on cerium oxide prepared using mechanochemical methods (planetary ball milling) can be as active or even more active than conventional catalysts prepared by incipient wetness impregnation for carbon monoxide oxidation, with the added advantage that mechanochemical methods are faster, simpler, and more environmentally friendly. A particularly relevant aspect has been the study of mechanochemical synthesis conditions. A detailed investigation was carried out to assess their effect on catalytic activity using a design of experiments, varying the milling energy and atmosphere. The results indicate that milling energy plays a critical role, with the catalyst obtained at 250 rpm significantly outperforming other catalysts prepared within the range of 100 to 1000 rpm. Additionally, catalysts prepared under oxidizing atmospheres are more active than those prepared under inert or reducing atmospheres. Another important parameter is the milling time; the catalyst prepared at 250 rpm for 10 min proved more active than those prepared under the same conditions for 5 or 30 min. One of the most interesting findings of this study is the establishment of a relationship between the catalytic activity in the CO oxidation reaction and the presence of nanostructured, reactive PdO_*x*_-Ce species, identified and quantified via XPS. A direct correlation was found between the amount of these species and catalytic activity. Even the catalyst prepared by incipient wetness impregnation, which initially contained a lower amount of active PdO_*x*_-Ce species, became activated over time and ultimately matched the activity of the catalyst prepared mechanochemically at 250 rpm for 10 min in air. After 70 h of reaction, both catalysts exhibited a similar amount of PdO_*x*_-Ce species. These species have been correlated with the presence of palladium nanoclusters observed by HAADF-STEM on the cerium oxide surface, showing a strong metal–support interaction, consistent with H_2_-TPR results. Through this study, we aimed to illustrate an example of the potential of mechanochemistry in the preparation of supported metal catalysts, emphasizing the influence of various parameters, particularly the atmosphere, a factor that has been little studied in this field. Furthermore, we have highlighted that this method generates novel nanoarchitectures on the surface of catalysts that are active in catalysis.

## Supplementary Material



## Notes

CO conversion curves; adsorption and desorption isotherms; Raman spectra; XPS spectra and surface elemental composition; SEM images; HRTEM images; design of experiments factors and mean S/N ratios; and TPR data (PDF)
