# Supplementary material for: Pd Supported on CeO2 Nanostructures Prepared by Planetary Ball Milling under a Modified Atmosphere for Catalytic Oxidation of CO
Source: ACS Appl Nano Mater. 2025 Jun 4;8(23):12151–63. doi: 10.1021/acsanm.5c01769 (PMC12172315; doi:10.1021/acsanm.5c01769)
Supplement: Supplementary file 1 [file an5c01769_si_001.pdf]

Supporting Information

Pd Supported on CeO_2 Nanostructures Prepared by Planetary Ball Milling under a Modified Atmosphere for Catalytic Oxidation of CO

Enrique Marín[a], Xavier Vendrell[b,], Jordi Llorca[a,*]*

[a] Institute of Energy Technologies, Department of Chemical Engineering and Center for Research in Multiscale Science and Engineering, Universitat Politècnica de Catalunya, EEBE, Eduard Maristany 10-14, 08019 Barcelona, Spain

[b] Department of Inorganic & Organic Chemistry and Institute of Nanoscience and Nanotechnology (IN2UB), Universitat de Barcelona, 08028 Barcelona, Spain

Corresponding Author: Jordi Llorca (jordi.llorca@upc.edu)

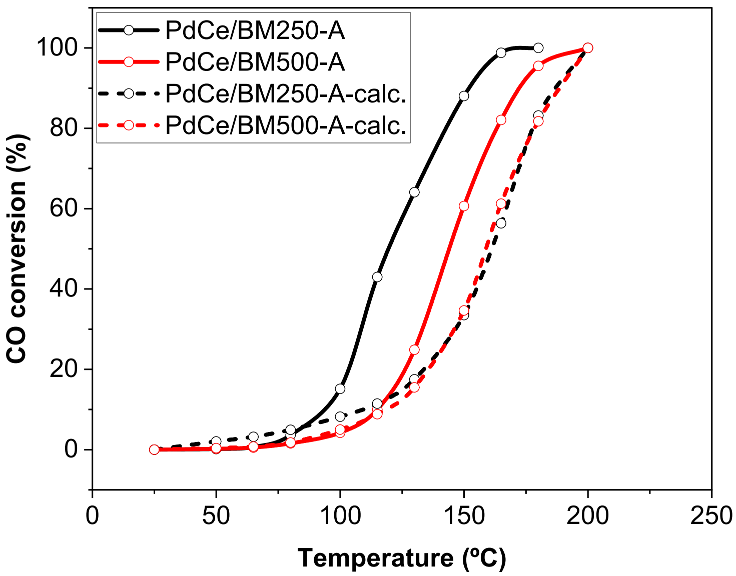

Figure S1. CO conversion curves for Pd/CeO_2 catalysts prepared using ball milling at 250 rpm and 500 rpm in air before and after calcination at 450 °C for 4 hours.

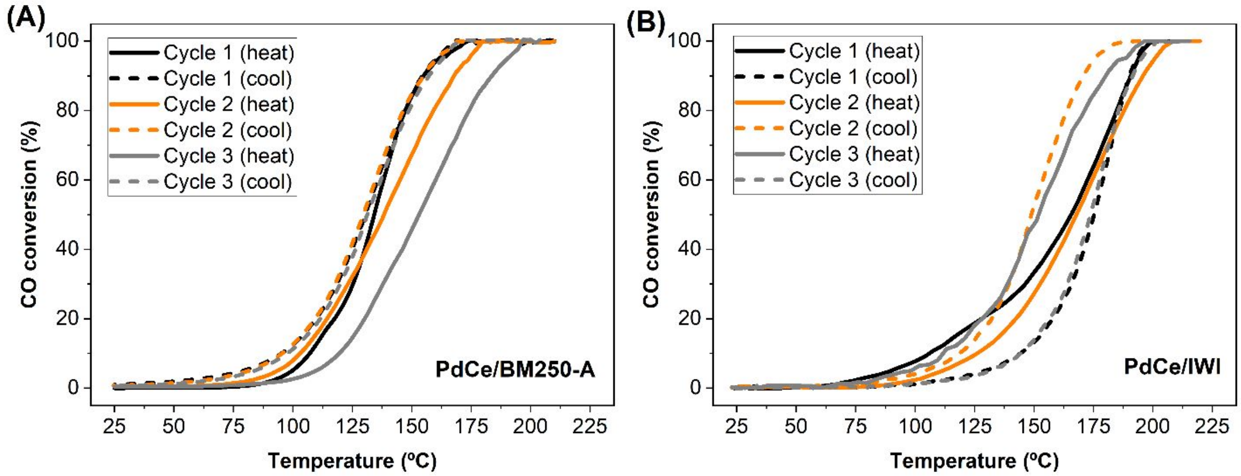

Figure S2. CO conversion during thermal cycling tests of PdCe/BM250-A (A) and PdCe/IWI (B) subjected to three cycles of heating (solid lines) and cooling (dotted-lines) between 25 and 225 °C.

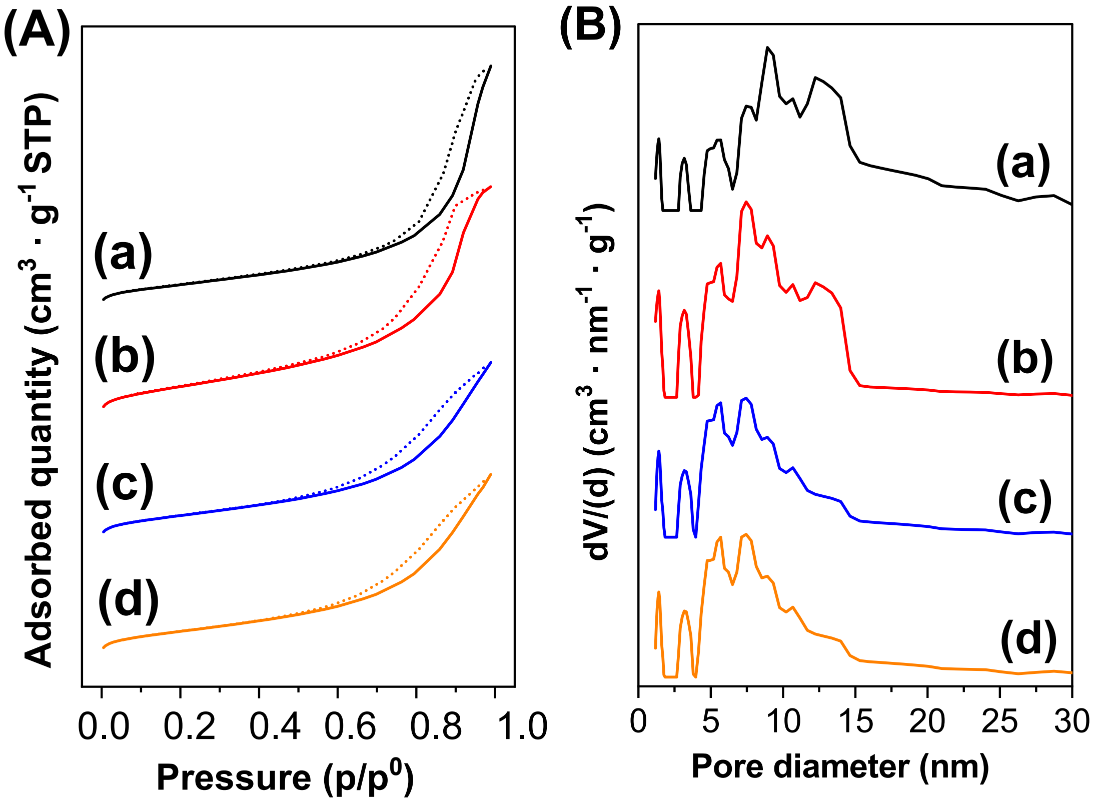

Figure S3. (A) N_2-physisorption measurements at 77 K: adsorption (solid lines) and desorption (dotted lines) isotherms, (B) pore size distribution. (a) bare ceria and Pd/CeO_2 catalysts prepared by (b) incipient wetness impregnation and ball milling at (c) 250 rpm and (d) 850 rpm in air.

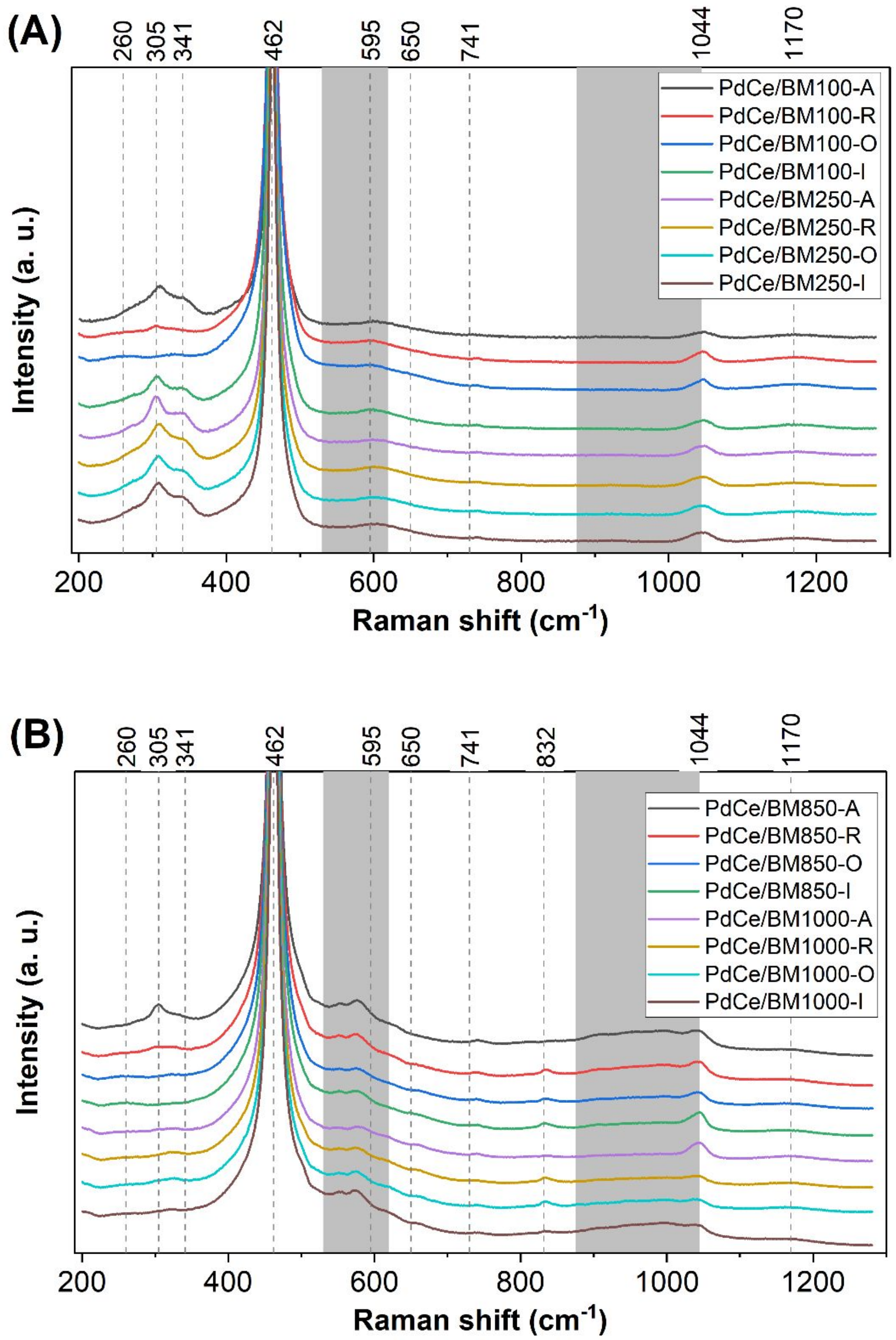

Figure S4. (A) Raman spectra of Pd/CeO_2 catalysts prepared by ball milling at 100 and 250 rpm, and (B) 850 and 1000 rpm under different atmospheres.

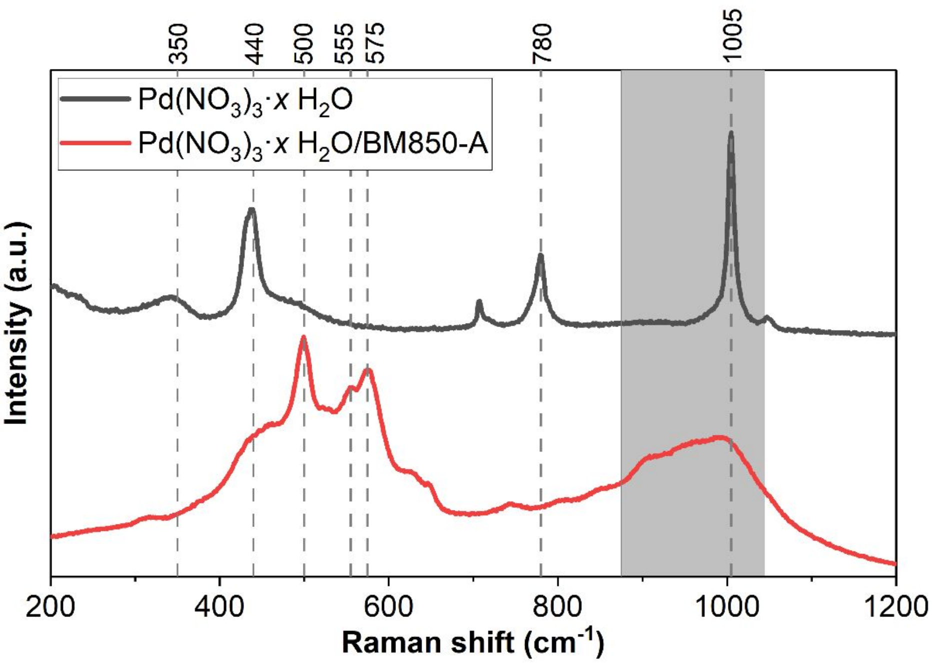

Figure S5. Raman spectra of the precursor $Pd(NO_3)_2xH_2O$ before and after ball milling in air at 850 rpm.

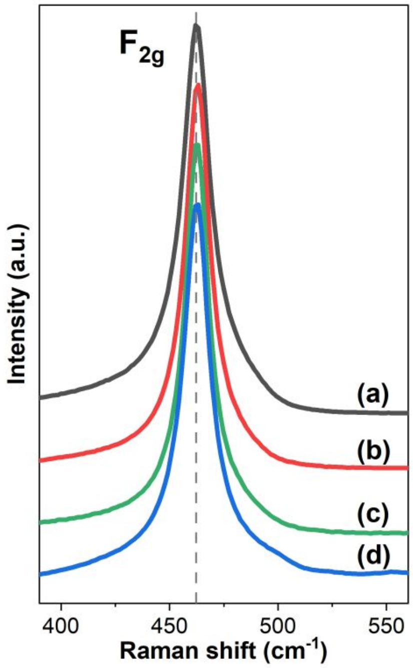

Figure S6. F_{2g} band in Raman spectra of bare ceria (a) and Pd/CeO_2 catalysts prepared by the IWI method (b), and ball milling in air at 250 rpm (c) and 850 rpm (d).

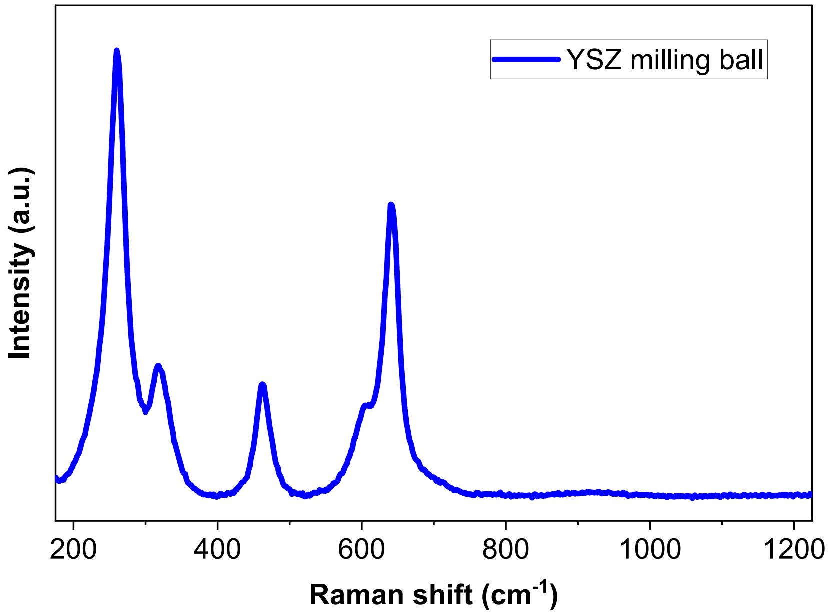

Figure S7. Raman spectra of a YSZ milling ball

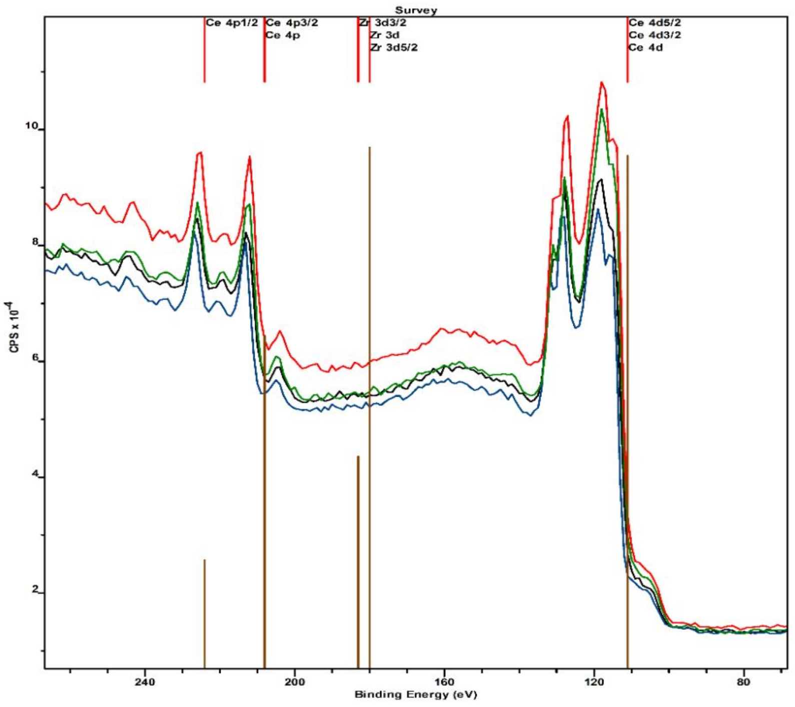

Figure S8. XPS survey spectrum indicating the absence of the most intense peaks associated with Zr 3d. The data corresponds to bare ceria (green), PdCe/IWI (red), PdCe/BM250-A (black), and PdCe/BM850-A (blue) samples. Bare ceria was not subjected to any milling treatment.

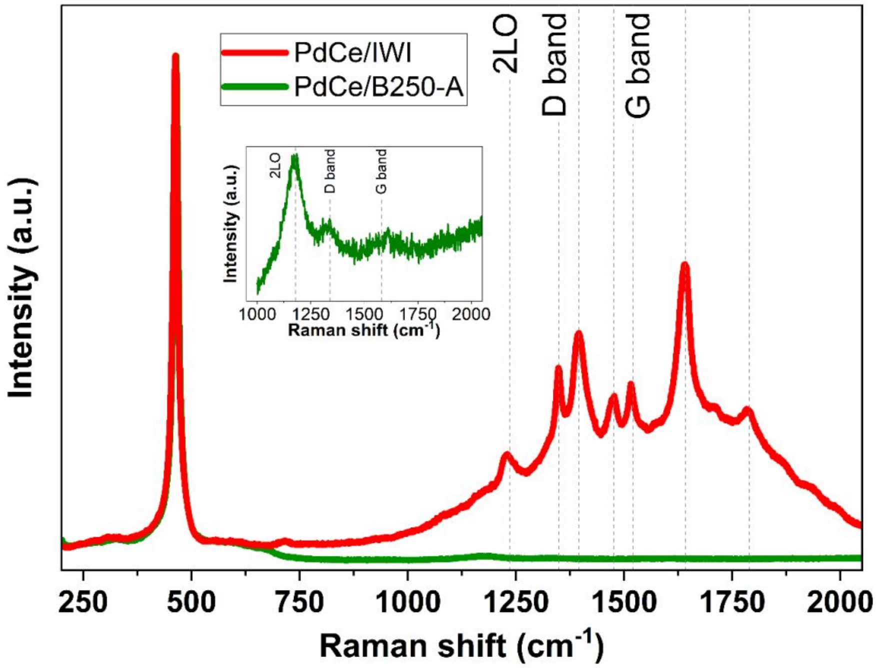

Figure S9. Raman spectra of Pd/CeO_2 catalysts after the CO oxidation reaction, prepared by incipient wetness impregnation and ball milling at 250 rpm under ambient conditions. The spectra correspond to PdCe/BM250-A sample subjected to a 70-hour stability test at 150 ˚C, and to PdCe/IWI sample subjected to a thermal cycling test. The inset highlights the region from 1000 to 2000 cm^{-1} for the PdCe/BM250-A sample.

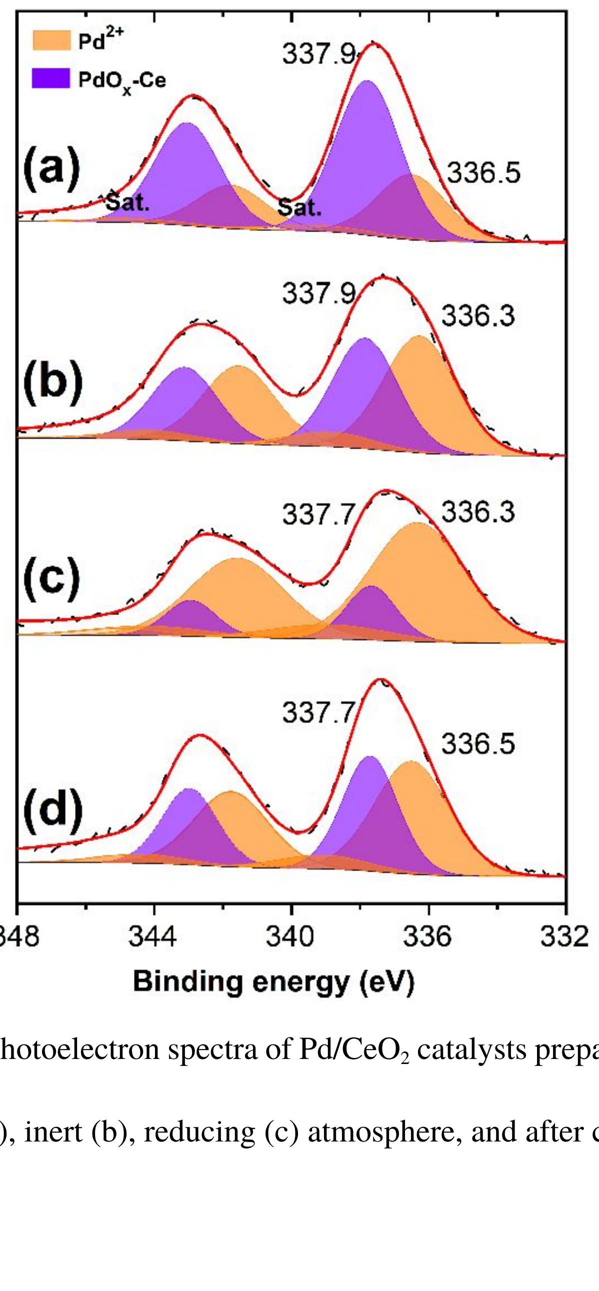

Figure S10. Pd 3d X-ray photoelectron spectra of Pd/CeO_2 catalysts prepared by ball milling in air at 250 rpm at oxidizing (a), inert (b), reducing (c) atmosphere, and after calcination at 450 ºC (d).

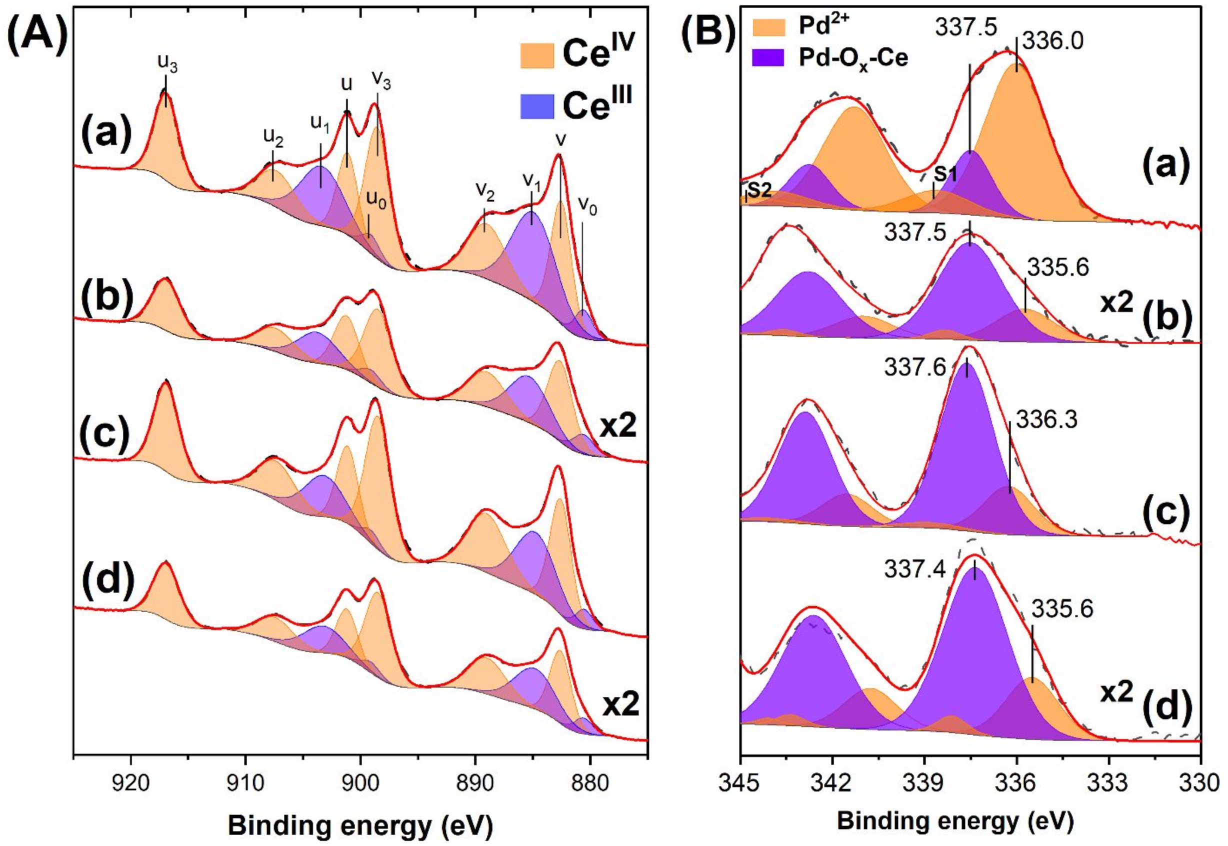

Figure S11. (A) Ce 3d and (B) Pd 3d X-ray photoelectron spectra of Pd/CeO_2 catalysts prepared by incipient wetness impregnation: fresh and after reaction (a and b), and ball milling in air at 250 rpm: fresh and after reaction (c and d).

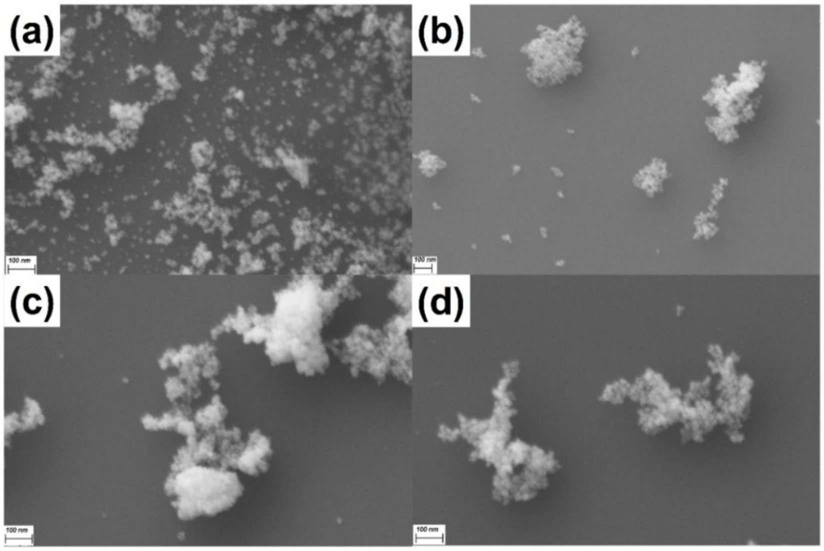

Figure S12. SEM micrographs of bare ceria (a) and Pd/CeO_2 catalysts prepared by the IWI method (b), and ball milling in air at 250 rpm (c) and 850 rpm (d).

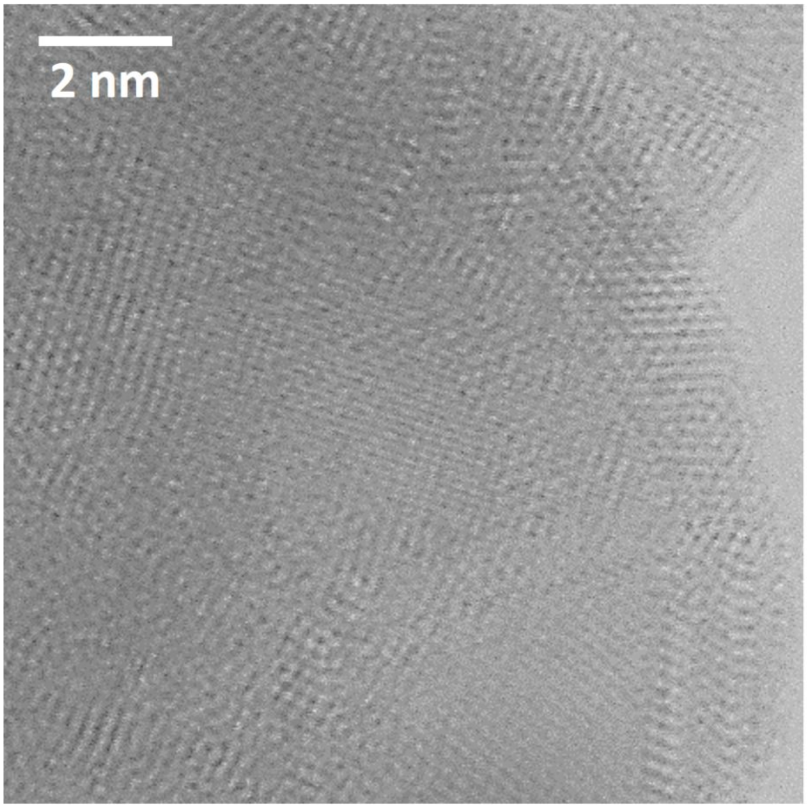

Figure S13. HRTEM image of the Pd/CeO_2 catalyst prepared by ball milling at 850 rpm in air.

Table S1. Control factors and levels for full factorial experimental analysis.

**Factors**	**Levels**			
	**1**	**2**	**3**	**4**
**Atmosphere**	Normal	Oxidizing	Inert	Reducing
**Milling velocity**	100	250	500	1000

Table S2. Design of experiments for a L16 (4^2) full factorial design and responses at different temperatures in CO oxidation reaction.

Experiment Nº	Control factors		Responses: CO_2 production ($mmol\ CO_2 \cdot g\ Pd^{-1} \cdot h^{-1}$) at		
	Atmosphere	Milling velocity (rpm)	115 ºC	150 ºC	180 ºC
1	Ambient	100	579	2106	2725
2	Oxidizing	250	1940	4273	5197
3	Inert	500	477	3246	5019
4	Reducing	1000	325	1246	3566
5	Oxidizing	100	265	1274	5361
6	Inert	250	1409	3683	5142
7	Reducing	500	555	2603	4589
8	Ambient	1000	111	569	2129
9	Inert	100	302	1175	2306
10	Reducing	250	469	2557	3997
11	Ambient	500	380	1952	4546
12	Oxidizing	1000	263	1105	3185
13	Reducing	100	285	1229	3124
14	Ambient	250	2304	4756	5361
15	Oxidizing	500	553	2769	5103
16	Inert	1000	363	1117	2758

Table S3. Absolute errors in °C for T_{20}, T_{50}, and T_{90} values for samples milled at different velocities and for the conventional sample prepared by the IWI method and by using a mortar and pestle.

Temperature (ºC)	Milling velocity (rpm)						IWI	Mortar
	100	125	250	500	850	1000		
**T20**	19.0	2.2	3.6	2.3	1.3	1.3	6.0	3.1
**T50**	22.6	7.1	3.4	3.0	2.4	1.5	2.1	8.1
**T90**	21.1	8.5	4.0	3.3	2.1	1.5	4.4	2.9

Table S4. Mean signal-to-noise ratios.

	Levels	Atmosphere	Milling velocity
**Mean S/N ratio**	1	57.93	55.05
	2	58.65	66.15
	3	58.65	58.30
	4	56.42	52.14
**Delta**		2.22	14.01
**Rank**		2	1

Table S5. Textural properties of the CeO_2 support and the Pd/CeO_2 catalysts

Sample	SSA_{BET} ($m^2\ g^{-1}$) [a]	Total pore volume ($cm^3\ g^{-1}$)	Mesopores volume ($cm^3\ g^{-1}$)	Average pore diameter (nm) [b]
**CeO_2**	49	0.170	0.149	8.92
**PdCe/IWI**	52	0.123	0.118	7.45
**PdCe/BM250-A**	50	0.124	0.107	7.45
**PdCe/BM850-A**	50	0.130	0.110	7.45

[a] Specific surface area calculated by BET method
[b] Average pore diameter calculated from DFT method

Table S6. I_D/F_{2g} ratio, lattice parameter and crystallite size for CeO_2 and Pd/CeO_2 catalysts.

Sample	CeO_2 lattice parameter a (Å)	Crystallite size, τ [b] (nm)	I_D/I_{F2g} [c]
**CeO_2**	5.421 ± 0.003	12.9 ± 0.6	0.024
**PdCe/IWI**	5.422 ± 0.003	12.9 ± 0.7	0.080
**PdCe/BM250-A**	5.417 ± 0.001	12.8 ± 0.7	0.054
**PdCe/BM850-A**	5.431 ± 0.005	12.3 ± 0.6	0.077

[a, b] calculated from Scherrer equation and (111), (200), (220) and (311) reflections
[c] calculated from Raman spectra

Table S7. Surface elemental composition measured by XPS in 250 rpm-milled samples under different atmospheres.

Sample	Pd^{2+} (%)	PdO_x-Ce (%)	Pd/Ce[a]	T50 (^oC)
**PdCe/BM250-A**	24	76	0.057	120
**PdCe/BM250-O**	35	65	0.069	125
**PdCe/BM250-I**	58	42	0.083	135
**PdCe/BM250-R**	83	17	0.071	152
**PdCe/BM250-C**	61	39	0.098	160

[a] Surface atomic ratio

Table S8. Summary of TPR results.

Sample	H_2 uptake (μmol/g_{cat}) of low-temperature peak[a]	H_2 uptake (μmol/g_{cat}) of surface CeO_2 peak	H_2 uptake (μmol/g_{cat}) of bulk CeO_2 peak
**CeO_2**	0	230	604
**PdCe/IWI**	565 (94)	1119	446
**PdCe/BM250-A**	1342 (376)	1518	590
**PdCe/BM850-A**	1234 (376)	1316	609

[a] Theoretical value is in parenthesis
